# Alkali-Metal Complexes of XeF_2_: Synthesis and Structural Characterization of [Li(XeF_2_)_3_PtF_6_], [Na(XeF_2_)PtF_6_], and [K(XeF_2_)_2_PtF_6_]

**DOI:** 10.1021/acs.inorgchem.6c04065

**Published:** 2026-10-11

**Authors:** Klemen Motaln, Dominik Kurzydłowski, Matic Lozinšek

**Affiliations:** † Extreme Conditions Chemistry Laboratory, Jožef Stefan Institute, Jamova Cesta 39, 1000 Ljubljana, Slovenia; ‡ Jožef Stefan International Postgraduate School, Jamova Cesta 39, 1000 Ljubljana, Slovenia; § Faculty of Mathematics and Natural Sciences, Cardinal Stefan Wyszyński University, ul. Wóycickiego 1/3, 01-938 Warsaw, Poland

## Abstract

Reactions of alkali-metal hexafluoridoplatinates­(V), MPtF_6_ (M = Li, Na, K, Rb), with xenon difluoride in anhydrous hydrogen fluoride were investigated. These reactions afforded the XeF_2_ coordination compounds [Li­(XeF_2_)_3_PtF_6_], [Na­(XeF_2_)­PtF_6_], and [K­(XeF_2_)_2_PtF_6_], whereas no Rb^+^ complex was isolated. The products were characterized by low-temperature single-crystal X-ray diffraction and Raman spectroscopy, complemented by periodic density functional theory calculations. The alkali-metal coordination number increases progressively from six for Li^+^ to seven for Na^+^ and eight for K^+^. This series expands the currently limited set of XeF_2_ complexes with monovalent cations and provides the first example of XeF_2_ coordination to K^+^ as well as the first monovalent cation with a homoleptic XeF_2_ coordination sphere, [Li­(XeF_2_)_6_]^+^, observed in [Li­(XeF_2_)_3_PtF_6_]. In addition, single-crystal structures and Raman spectra of the parent salts LiPtF_6_, NaPtF_6_, KPtF_6_, and RbPtF_6_ are reported.

## Graphical abstract

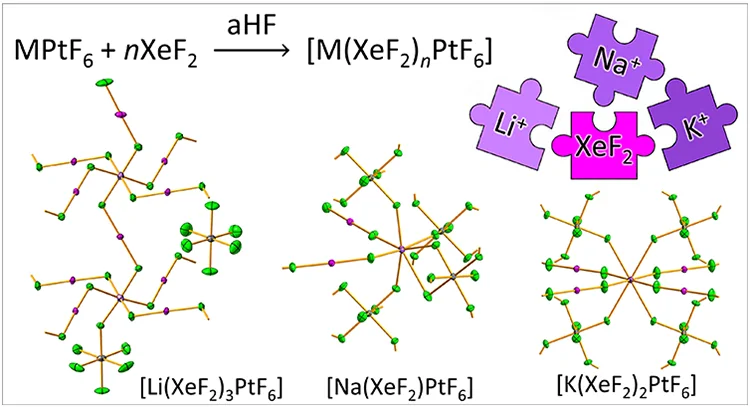

## Introduction

Owing to its Lewis basicity,^1^ XeF_2_ readily acts as a ligand toward “naked” metal cations in salts of weakly coordinating anions, and the resulting coordination complexes of the general type [M^*x*+^(XeF_2_)_*n*_(AF_6_^–^)_*x*_] (*x* = 1, 2, 3; 1 ≤ *n* ≤ 8; A = P, V, As, Nb, Ru, Sb, Ta, Ir, Bi) constitute a large and structurally diverse family of noble-gas compounds.^2–6^ The first such compound, [Ag­(XeF_2_)_2_AsF_6_], was obtained by oxidation of gaseous Xe with a solution of AgFAsF_6_ in anhydrous HF (aHF).^7^ Since then, numerous XeF_2_ coordination complexes involving monovalent,^7–9^ divalent,^10–15^ and trivalent metal centers^16–18^ have been prepared, and noble-gas fluoride coordination chemistry has been extended to XeF_4_ and KrF_2_ ligands,^19,20^ and to tetravalent fluoridometal complexes.^21^ Although a relatively large number of structures involving coordination of XeF_2_ to divalent and trivalent metal cations have been reported,^5,6^ only five complexes featuring monovalent cations, including Li^+^, Na^+^, and Ag^+^, have been crystallographically characterized. This scarcity is attributable to the oxidizing power of XeF_2_ in aHF,^22^ which leaves only a limited subset of oxidation-resistant monovalent metal cations as viable candidates for ligation by XeF_2_. In this context, alkali-metal cations are a suitable choice for preparation of novel coordination compounds. Investigations of LiAF_6_–XeF_2_ (A = P, As, V, Ru, Ir, Sb, Nb, Ta) and MAsF_6_–XeF_2_ (M = Li, Na, K, Rb, Cs) systems afforded coordination complexes with Li^+^, [Li­(XeF_2_)_*n*_AF_6_] (A = P, As, Ru, Ir). Of these, only [Li­(XeF_2_)_3_AsF_6_] was structurally elucidated. All the Li^+^ complexes decomposed at ambient temperature, and no coordination compounds of the heavier alkali metals were isolated.^9^ By contrast, reactions of XeF_2_ with alkali-metal fluoridooxidovanadates­(V) afforded the Li^+^ and Na^+^ complexes [Li_2_(XeF_2_)­(V_2_O_2_F_8_)] and [Na_2_(XeF_2_)­(V_2_O_2_F_8_)], which do not lose XeF_2_ at ambient temperature.^23,24^ Beyond the formation of isolable coordination compounds, the XeF_2_–Li^+^ interaction plays a role in the recently described activation of XeF_2_ by the lithium salt Li­[Al­{OC­(CF_3_)_3_}_4_], thereby enabling the oxidative coupling of (fluoro)­benzenes and the formation of (fluorinated) biphenyl radical cations.^25^

In this study, the role of XeF_2_ as a ligand for alkali-metal cations was reexamined by investigating its reactions with alkali-metal hexafluoridoplatinates­(V) in aHF. This approach afforded [Li­(XeF_2_)_3_PtF_6_], [Na­(XeF_2_)­PtF_6_], and [K­(XeF_2_)_2_PtF_6_], which were characterized by low-temperature single-crystal X-ray diffraction (SCXRD) and Raman spectroscopy, together with periodic density functional theory (DFT) calculations. Notably, [K­(XeF_2_)_2_PtF_6_] constitutes the first example of XeF_2_ coordinated to a K^+^ cation, thereby extending the range of monovalent metal cations known to coordinate XeF_2_ and underscoring the importance of the anion selection in accessing previously unattainable XeF_2_ complexes. In addition, the SCXRD structures and Raman spectra of the parent salts LiPtF_6_, NaPtF_6_, KPtF_6_, and RbPtF_6_ are reported.

## Results and Discussion

### Syntheses

The starting alkali-metal hexafluoridoplatinates­(V) were synthesized in h-shaped reaction vessels comprising a straight main arm and an angled side arm (see Experimental section) in anhydrous HF (aHF) by direct oxidation of Pt powder with F_2_ under UV irradiation from an LED light source in the presence of the corresponding alkali-metal fluoride (eq 1).^26^

1 $$2\mathrm{MF}+2\mathrm{Pt}+5F_{2}\overset{\mathrm{UV},\mathrm{aHF}}{\to }2{\mathrm{MPtF}}_{6},M=\mathrm{Li},\mathrm{Na},K,\mathrm{Rb}$$

During the syntheses, it was observed that the formed LiPtF_6_ and NaPtF_6_ fully dissolved in aHF, affording bright orange-yellow solutions, whereas KPtF_6_ and RbPtF_6_ did not dissolve fully, leaving a small amount of yellow precipitate beneath an orange-yellow supernatant. After solvent removal, the MPtF_6_ salts were isolated as yellow crystalline solids.

To explore the coordination of XeF_2_ to the alkali-metal cations, the MPtF_6_ (M = Li, Na, K, Rb) salts were reacted with approximately 3 to 4 molar equivalents of XeF_2_ in aHF at room temperature (eq 2).

$$\begin{array}{l} {\mathrm{MPtF}}_{6}+m{\mathrm{XeF}}_{2}\overset{\mathrm{aHF}}{\to }\left[M{\left({\mathrm{XeF}}_{2}\right)}_{n}{\mathrm{PtF}}_{6}\right]+\left(m-n\right){\mathrm{XeF}}_{2},M=\mathrm{Li},\mathrm{Na},K;m=\mathrm{3-4};n=1,2,3 \end{array}$$ 2

Upon reaction of the alkali hexafluoridoplatinates­(V) with excess XeF_2_ in aHF, the solutions retained the same coloration as before the addition of XeF_2_, and a yellow precipitate persisted in the KPtF_6_ and RbPtF_6_ reaction mixtures. After a brief period of magnetic stirring, solutions were decanted from the main into the side arm of the reactors, and crystallization from the solutions was achieved by slow evaporation of the solvent. Once crystals had formed, the products were isolated by removing the solvent under dynamic vacuum. Because the isolated XeF_2_ coordination complexes were suspected to be unstable at ambient temperature, the products were cooled in an EtOH bath maintained below −15 °C during solvent removal.

Following this procedure, the coordination of XeF_2_ to Li^+^, Na^+^, and K^+^ was achieved, affording [Li­(XeF_2_)_3_PtF_6_], [Na­(XeF_2_)­PtF_6_], and [K­(XeF_2_)_2_PtF_6_], respectively. No evidence for the ligation of Rb^+^ by XeF_2_ was observed under these reaction conditions. Once isolated, the XeF_2_ coordination compounds were stored in a glovebox freezer maintained between −5 and −10 °C. Under these conditions, crystals of [Li­(XeF_2_)_3_PtF_6_] and [Na­(XeF_2_)­PtF_6_] retained XeF_2_ for more than six months, whereas [K­(XeF_2_)_2_PtF_6_] decomposed into XeF_2_ and KPtF_6_. Raman spectra of the insoluble precipitates remaining in the main arms in the KPtF_6_ and RbPtF_6_ reactions showed only bands attributable to the respective starting alkali hexafluoridoplatinate­(V) salts.

Analysis of the solids recovered from the side arms of the LiPtF_6_–XeF_2_ and RbPtF_6_–XeF_2_ reactions by SCXRD and Raman spectroscopy revealed that, in addition to the [Li­(XeF_2_)_3_PtF_6_] complex, RbPtF_6_ and unreacted XeF_2_, [Xe_2_F_3_]­[PtF_6_]·XeF_2_(*oP*120) had also formed.^27^ Its presence could be explained by the establishment of an equilibrium between the [Xe_2_F_3_]^+^ salt and the XeF_2_ coordination compound, accompanied by the release of the alkali fluoride (eq 3). Similar equilibria have been reported for the Cu­[RuF_6_]_2_–XeF_2_, Cu­[SbF_6_]_2_–XeF_2_, and Zn­[SbF_6_]_2_–XeF_2_ systems.^13–15^

$$\begin{array}{l} {\mathrm{MPtF}}_{6}+m{\mathrm{XeF}}_{2}\overset{\mathrm{aHF}}{\to }a\left[M{\left({\mathrm{XeF}}_{2}\right)}_{n}{\mathrm{PtF}}_{6}\right]+b\left[{\mathrm{Xe}}_{2}F_{3}\right]\left[{\mathrm{PtF}}_{6}\right]\cdot {\mathrm{XeF}}_{2}+c\mathrm{MF},M=\mathrm{Li},\mathrm{Rb}\;\left(a=0\right) \end{array}$$ 3

### Crystal Structures of XeF_2_ Coordination Complexes

### Crystal Structure of [Li­(XeF_2_)_3_PtF_6_]

[Li­(XeF_2_)_3_PtF_6_] crystallizes in the monoclinic space group *P*2_1_ (Tables 1, S1), as does the previously characterized [Li­(XeF_2_)_3_AsF_6_].^9^ Their unit cells are closely related: the *a* and *c* parameters are comparable, whereas the *b* parameter of [Li­(XeF_2_)_3_PtF_6_] is approximately twice that of the As analog. Consistent with this cell doubling, the Pt compound contains two symmetry-inequivalent Li^+^ sites (*Z*′ = 2) (Figure 1), whereas the As complex contains only one. Both Li^+^ cations in [Li­(XeF_2_)_3_PtF_6_] are approximately octahedrally coordinated (*cis*-F–Li–F: 84.6(3)–98.1(4)°; *trans*-F–Li–F: 172.0(5)–179.1(5)°; Tables 2, S2). One Li^+^ site is coordinated exclusively by six XeF_2_ ligands, one terminal and five bridging, forming the homoleptic [Li­(XeF_2_)_6_]^+^ coordination sphere—the first such motif for a monovalent metal cation. The second Li^+^ site is coordinated by five bridging XeF_2_ ligands and one [PtF_6_]^−^ anion. This environment resembles that in [Li­(XeF_2_)_3_AsF_6_], where Li^+^ is likewise coordinated by five XeF_2_ ligands and one [AsF_6_]^−^ anion. The sole Li–F­(Pt) bond (1.927(8) Å) is shorter than the Li–F­(Xe) bonds (1.943(9)–2.032(8) Å). Bridging XeF_2_ molecules link the Li^+^ cations into double layers parallel to the *ac* plane. Each double layer comprises pairs of Li^+^ sites stacked along *b*, with further propagation in this direction terminated by a terminal XeF_2_ ligand at one end and a Li^+^-coordinating [PtF_6_]^−^ anion at the other (Figure S1). All six crystallographically independent XeF_2_ molecules remain nearly linear (F–Xe–F: 177.68(10)–179.87(13)°). The terminal XeF_2_ ligand exhibits pronounced bond-length asymmetry, with a shorter terminal Xe–F_t_ bond (1.967(4) Å) and a longer coordinating Xe–F­(Li) bond (2.020(3) Å). Similar Xe–F bond lengths were reported for XeF_2_ in the hydrogen-bonded salt cocrystal CF_3_C­(OH)­NH_2_[AsF_6_]·XeF_2_.^28^ The Xe–F­(Li) bond lengths in the bridging XeF_2_ ligands differ by less than 3σ from the Xe–F bond length in free XeF_2_. Charge balance is provided by two symmetry-inequivalent [PtF_6_]^−^ anions, one of which coordinates Li^+^, whereas the other does not participate in Li^+^ coordination and occupies voids in the structure (Figures 1, S1). Both anions retain nearly ideal octahedral geometries (*cis*-F–Pt–F: 88.37(17)–90.78(16)°; *trans*-F–Pt–F: 178.78(15)–179.8(2)°). The terminal Pt–F_t_ bonds (1.868(4)–1.889(4) Å) are shorter than the Li^+^-coordinating Pt–F­(Li) bond (1.909(3) Å).

**Table 1:** Summary of Crystallographic Data and Refinement Results for [Li­(XeF_2_)_3_PtF_6_], [Na­(XeF_2_)­PtF_6_], and [K­(XeF_2_)_2_PtF_6_] at 100 K

	[Li(XeF_2_)_3_PtF_6_]	[Na(XeF_2_)PtF_6_]	[K(XeF_2_)_2_PtF_6_]
Space group	*P*2_1_	*P*2_1_/*c*	*I*4/*mcm*
*a* (Å)	6.80526(6)	7.1459(3)	8.73777(9)
*b* (Å)	26.79621(19)	10.0427(4)	8.73777(9)
*c* (Å)	6.81133(6)	10.2322(4)	13.3131(2)
α (°)	90	90	90
β (°)	92.6673(8)	95.852(4)	90
γ (°)	90	90	90
*V* (Å^3^)	1240.736(17)	730.47(5)	1016.44(3)
*Z*	4	4	4
*F*_w_ (g mol^–1^)	823.93	501.38	686.79
ρ_calc_ (g cm^–3^)	4.411	4.559	4.488
μ (mm^–1^)	10.363	12.918	11.227
*R*[*F* ^2^ > 2σ(*F* ^2^)]	0.0284	0.0358	0.0174
*w*R** (*F* ^2^)	0.0706	0.1042	0.0495
Δρ_min_, Δρ_max_ (e Å^–3^)	–3.231, 3.445	–3.226, 3.440	–1.576, 1.525

**Table 2:** Selected Bond Lengths (Å) and Angles (°) for [Li­(XeF_2_)_3_PtF_6_], [Na­(XeF_2_)­PtF_6_], and [K­(XeF_2_)_2_PtF_6_] Complexes^a^

	[Li(XeF_2_)_3_PtF_6_]	[Na(XeF_2_)PtF_6_]	[K(XeF_2_)_2_PtF_6_]
Xe–F_t_	1.967(4)	-	-
Xe–F(M)	1.994(2)–2.020(3)	1.977(2), 1.997(2)	1.9829(10)
Pt–F_t_	1.868(4)–1.889(4)	1.864(2)	1.8766(18)
Pt–F(M)	1.909(3)	1.880(2)–1.897(2)	1.8787(9)
M–F(Xe)	1.943(9)–2.032(8)	2.250(3), 2.254(3)	2.6121(10)
M–F(Pt)	1.927(8)	2.292(3)–2.910(3)	2.7542(9)
F–Xe–F	177.68(10)–179.87(13)	179.03(12)	180.0
*cis*-F–Pt–F	88.37(17)–90.78(16)	87.57(12)–92.21(12)	90.0
*trans*-F–Pt–F	178.78(15)–179.8(2)	178.98(12)–179.65(11)	180.0

a M denotes Li, Na, or K. F_t_ denotes a terminal F atom.

**Figure 1:**
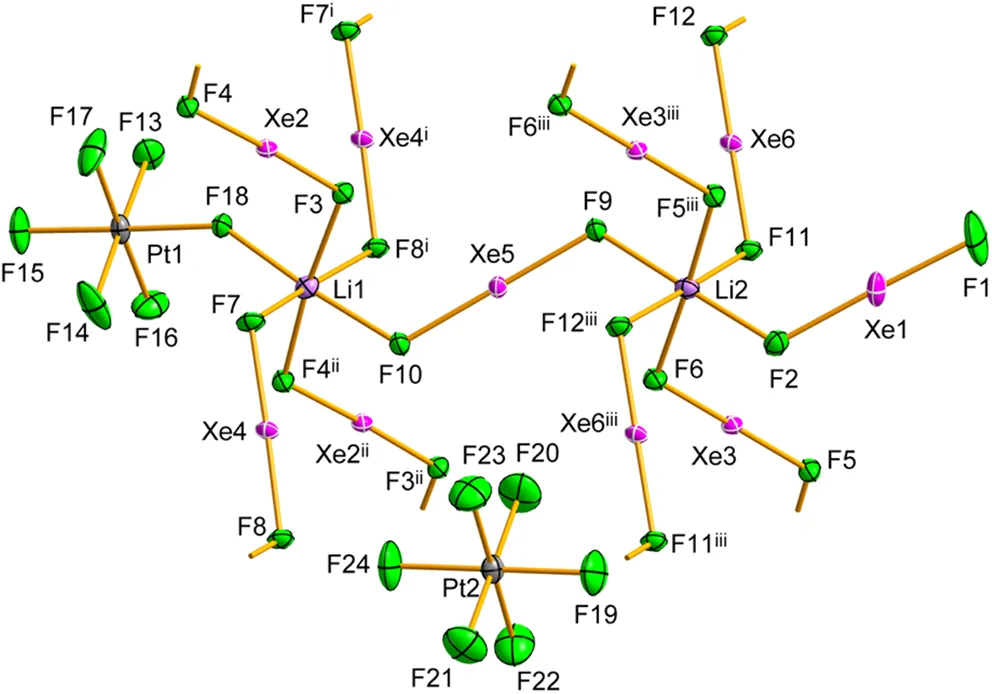
Expanded asymmetric unit of the crystal structure of [Li­(XeF_2_)_3_PtF_6_] showing the coordination environments of the two symmetry-inequivalent Li^+^ cations. Displacement ellipsoids are depicted at the 50% probability level. Symmetry codes used for generation of equivalent atoms: (i) *x*, *y*, *z* + 1; (ii) *x* + 1, *y*, *z*; (iii) −*x* + 1, *y* – 1/2, −*z* + 1.

### Crystal Structure of [Na­(XeF_2_)­PtF_6_]

[Na­(XeF_2_)­PtF_6_] crystallizes in the monoclinic space group *P*2_1_/*c* with one formula unit in the asymmetric unit (*Z*′ = 1) and all atoms in general positions (Tables 1, S1). The Na^+^ coordination sphere comprises seven F atoms in a distorted pentagonal-bipyramidal arrangement (Figure 2). Two F atoms from two XeF_2_ ligands form the shortest Na–F­(Xe) bonds (2.250(3) and 2.254(3) Å) (Tables 2, S3) and are mutually *cis*, with one occupying an axial and the other an equatorial position. Each XeF_2_ molecule bridges two adjacent Na^+^ cations, forming zigzag chains that propagate along the *c* axis (Figure S2). Despite being a bridging ligand, XeF_2_ has unequal Xe–F bonds (1.977(2) and 1.997(2) Å) but remains essentially linear (179.03(12)°). The remaining five coordination sites around Na^+^ are occupied by F atoms from four [PtF_6_]^−^ anions: three anions coordinate monodentately (2.292(3)–2.365(3) Å), whereas the fourth coordinates bidentately through one shorter and one substantially longer Na–F­(Pt) bond (2.336(3), 2.910(3) Å). Each [PtF_6_]^−^ anion coordinates four neighboring Na^+^ centers, extending the structure into a three-dimensional network (Figure S2). The [PtF_6_]^−^ anion adopts a slightly distorted octahedral geometry (*cis*-F–Pt–F: 87.57(12)–92.21(12)° and *trans*-F–Pt–F: 178.98(12)–179.65(11)°). The sole terminal Pt–F_t_ bond (1.864(2) Å) is notably shorter than the bridging Pt–F­(Na) bonds (1.880(2)–1.897(2) Å).

**Figure 2:**
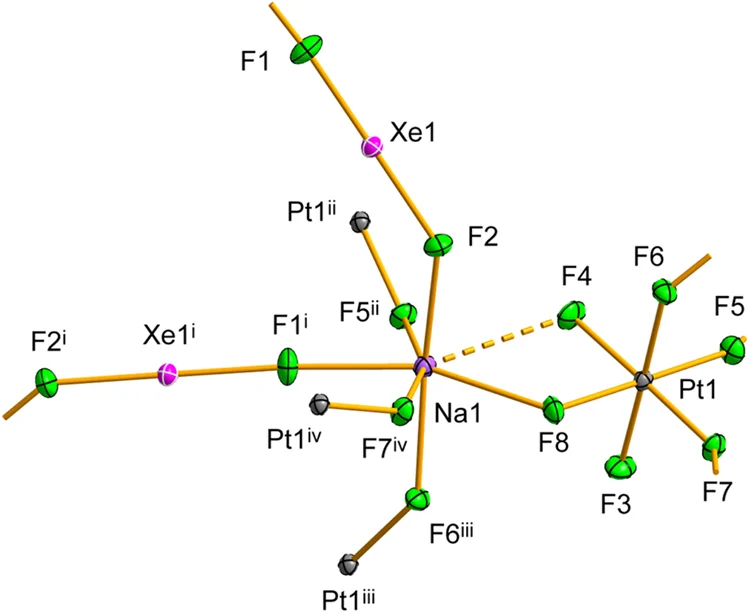
Coordination environment of Na^+^ in [Na­(XeF_2_)­PtF_6_]. Displacement ellipsoids are drawn at the 50% probability level. Symmetry codes used for generation of equivalent atoms: (i) *x*, −*y* + 1/2, *z* + 1/2; (ii) −*x* + 1, *y* – 1/2, −*z* + 3/2; (iii) *x*, −*y* + 3/2, *z* + 1/2; (iv) −*x*, *y* – 1/2, −*z* + 3/2.

### Crystal Structure of [K­(XeF_2_)_2_PtF_6_]

[K­(XeF_2_)_2_PtF_6_] crystallizes in the tetragonal space group *I*4/*mcm*, with all atoms residing on special positions (*Z*′ = 0.125) and unit-cell parameters [*a* = 8.73777(9) Å, *c* = 13.3131(2) Å, *V* = 1016.44(3) Å^3^] (Tables 1, S1) that closely resemble those of the previously characterized isostructural pair [Ag­(XeF_2_)_2_PF_6_] [*a* = 8.467(3) Å, *c* = 12.745(3) Å, *V* = 913.69(18) Å^3^][Bibr ref8] and [Ag­(XeF_2_)_2_AsF_6_] [*a* = 8.4558(12) Å, *c* = 12.8645(19) Å, *V* = 919.8(3) Å^3^].^7^ The larger unit-cell volume of the K^+^ complex is consistent with the bulkier [PtF_6_]^−^ anion. However, the structures of the Ag^+^ analogues were refined in the noncentrosymmetric space group *I*4̅*c*2, whereas the intensity-distribution statistics for [K­(XeF_2_)_2_PtF_6_] strongly support a centrosymmetric space group *I*4/*mcm*, which also yields a model with lower *R* indices. Nevertheless, the structure of [K­(XeF_2_)_2_PtF_6_] closely resembles those of the Ag^+^ complexes. The K^+^ cation is coordinated by eight F atoms in a slightly distorted cube shape, with four F atoms contributed by bridging XeF_2_ ligands and four by [PtF_6_]^−^ anions (Figures 3, S3). The K–F­(Xe) bonds (2.6121(10) Å) are shorter than the K–F­(Pt) bonds (2.7542(9) Å) (Tables 2, S4). Each XeF_2_ molecule bridges two K^+^ centers and retains ideal linear *D*_∞h_ symmetry, with equal Xe–F bond lengths (1.9829(10) Å) that are only slightly shorter than those in solid XeF_2_. The [PtF_6_]^−^ anion likewise retains nearly ideal octahedral geometry (*cis*-F–Pt–F: 90°, *trans*-F–Pt–F: 180°), with four equivalent equatorial Pt–F­(K) bonds (1.8787(9) Å) and two apical Pt–F_t_ bonds (1.8766(18) Å). The K–F­(Pt) interactions generate layers of K and Pt atoms in a NaCl-type arrangement parallel to the *ab* plane, while the bridging XeF_2_ molecules connect adjacent layers along *c*, producing a three-dimensional network (Figure S4).

**Figure 3:**
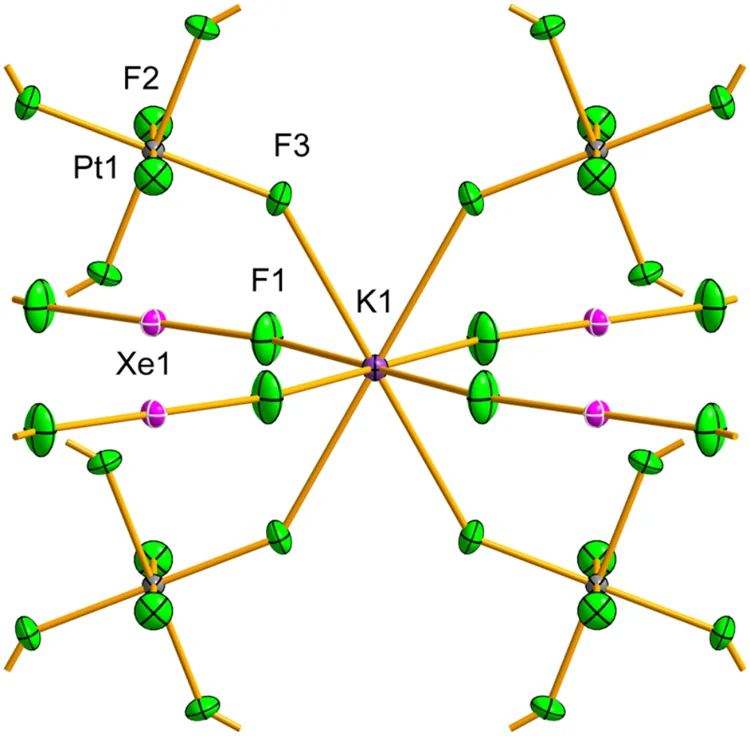
Coordination environment of K^+^ in the [K­(XeF_2_)_2_PtF_6_] complex. Displacement ellipsoids are drawn at the 50% probability level.

### Computational Results

The geometries of the alkali-metal XeF_2_ coordination complexes were optimized using periodic density functional theory (DFT) calculations at the PBEsol-TS and rSCAN-D4 levels of theory. The optimizations converged for all three structures, and the optimized geometries agree well with the experimental structures. Calculated bond lengths differ from the experimental values by no more than 3.5%, with the Xe–F bonds typically being overestimated by approximately 2% (Tables S2–S4).

The electronic structures of the complexes were further examined by calculating Hirshfeld atomic charges. At the PBEsol-TS level, the calculated charges across the three structures range from +1.09 to +1.16 e for Xe atoms and from +1.50 to +1.52 e for Pt, and from −0.58 to −0.38 e for F (Tables S5–S7). For comparison, the Hirshfeld charges in crystalline XeF_2_ calculated using the same method are +1.05 e for Xe and −0.53 e for F. Summation of the atomic charges for each XeF_2_ molecule shows that all XeF_2_ units in the three complexes carry a small net positive charge.

In [Li­(XeF_2_)_3_PtF_6_], the Xe atom of the sole terminal XeF_2_ ligand carries the lowest positive charge among the Xe atoms. The F atoms bonded to this Xe atom differ markedly in charge, with the terminal F atom (−0.45 e) bearing less negative charge than the bridging F atom (−0.57 e). These values bracket all other calculated charges for F atoms in XeF_2_ units, indicating greater polarization of the terminal ligand than of the bridging XeF_2_ molecules. Such polarization commonly arises from interactions between XeF_2_ and Lewis-acidic species and promotes partial ionization along the XeF_2_ → XeF^+^ + F^–^ pathway.^29,30^ The sole Li^+^-coordinating F atom of the [PtF_6_]^−^ anion is likewise more negatively charged (−0.58 e) than its noncoordinating F atoms (−0.43 to −0.40 e). Similar negative charges are also carried by the F atoms bonded to the Pt2 atom of the non-coordinated [PtF_6_]^−^ anion (−0.43 to −0.41 e). Similarly, in [Na­(XeF_2_)­PtF_6_], the terminal F3 atom and F4, which forms the longest Na–F­(Pt) contact, carry charges (−0.38 and −0.40 e, respectively), which are notably less negative than those calculated for the other Na^+^-coordinating F atoms (−0.51 to −0.46 e). By contrast, in [K­(XeF_2_)_2_PtF_6_] the noncoordinating F2 atom of [PtF_6_]^−^ is slightly more negatively charged (−0.48 e) than the K^+^-coordinating F3 atom (−0.45 e). The absence of increased negative charge at the K^+^-coordinating F atom may reflect the lower polarizing power of K^+^ relative to Li^+^ and Na^+^.

Moreover, the energetic favorability of XeF_2_ ligation was evaluated by calculating the static-lattice energy changes (Δ*E*) for the formation of the coordination complexes from crystalline XeF_2_ and the corresponding MPtF_6_ salts (Table S8 and Figure S5).

### Crystal Structures of MPtF_6_ Compounds (M = Li, Na, K, Rb)

Previous structural studies of MPtF_6_ salts (M = Li, K, Rb) were based on powder X-ray diffraction (PXRD). Only rhombohedral unit-cell parameters were reported for KPtF_6_ and RbPtF_6_,^31,32^ whereas LiPtF_6_ was characterized in greater detail at 299 K using synchrotron PXRD.^33^ Single-crystal structure determinations reported herein provide more detailed and precise structural models and reveal two distinct structure types: the lighter congeners LiPtF_6_ and NaPtF_6_ adopt the LiSbF_6_-type structure,^34^ whereas the heavier KPtF_6_ and RbPtF_6_ crystallize in the KAsF_6_-type structure.^35–37^ The latter two compounds are also isostructural with CsPtF_6_, whose structure was previously determined by SCXRD.^38^ All four compounds crystallize in the space group *R*3̅ with *Z* = 3 (Tables 3, 4, S9–S13). The experimentally observed structure-type preferences of the four salts, as well as the trend toward increasing unit-cell volume with increasing alkali-metal ionic radius, were also well reproduced by periodic DFT calculations (Tables S14, S15, Figure S6).

**Table 3:** Crystal Data and Refinement Results for the Structures of LiPtF_6_, NaPtF_6_, KPtF_6_, and RbPtF_6_ at 100 K

	LiPtF_6_	NaPtF_6_	KPtF_6_	RbPtF_6_
Space group	*R*3̅	*R*3̅	*R*3̅	*R*3̅
*a* (Å)	4.97492(11)	5.16973(7)	7.41013(13)	7.55677(11)
*b* (Å)	4.97492(11)	5.16973(7)	7.41013(13)	7.55677(11)
*c* (Å)	13.5916(3)	14.7717(2)	7.28292(15)	7.54000(12)
α (°)	90	90	90	90
β (°)	90	90	90	90
γ (°)	120	120	120	120
*V* (Å^3^)	291.321(15)	341.898(10)	346.329(14)	372.884(13)
*Z*	3	3	3	3
*F*_w_ (g mol^–1^)	316.03	332.08	348.19	394.56
ρ_calc_ (g cm^–3^)	5.404	4.839	5.008	5.271
μ (mm^–1^)	19.636	16.782	16.977	20.585
*R* [*F* ^2^ > 2σ(*F* ^2^)]	0.0192	0.0178	0.0183	0.0181
*w*R** (*F* ^2^)	0.0462	0.0413	0.0427	0.0436
Δρ_min_, Δρ_max_ (e Å^–3^)	–3.113, 2.805	–2.219, 3.295	–3.421, 2.138	–2.411, 2.573

**Table 4:** Bond Lengths (Å) and Angles (°) for LiPtF_6_, NaPtF_6_, KPtF_6_, and RbPtF_6_ ^a^

	LiPtF_6_	NaPtF_6_	KPtF_6_	RbPtF_6_
Pt–F	1.8841(15)	1.8842(12)	1.8855(13)	1.8843(11)
M–F	6 × 2.0179(15)	6 × 2.2899(13)	6 × 2.8369(14), 6 × 2.9367(15)	6 × 2.9290(13), 6 × 3.0600(13)
*cis*-F–Pt–F	6 × 89.78(7), 6 × 90.22(7)	6 × 89.52(6), 6 × 90.48(6)	6 × 88.07(7), 6 × 91.93(7)	6 × 88.61(6), 6 × 91.39(6)
*trans*-F–Pt–F	180.0	180.0	180.0	180.0

a M denotes Li, Na, K, or Rb.

In the isotypic LiPtF_6_ and NaPtF_6_ structures (Figure 4), each alkali-metal cation is coordinated by six F atoms in a slightly distorted octahedral geometry (Li–F: 2.0179(15) Å; Na–F: 2.2899(13) Å) (Table 4). For each cation, the six coordinating F atoms originate from six different [PtF_6_]^−^ anions, generating a three-dimensional network in which the alkali metal cations and Pt centers adopt a NaCl-like arrangement (Figure S7). The Pt–F bond length is essentially equal in both compounds (LiPtF_6_: 1.8841(15) Å; NaPtF_6_: 1.8842(12) Å) and agrees well with the previously reported value for LiPtF_6_ (1.887(6) Å).^33^ The [PtF_6_]^−^ anions in both compounds are essentially octahedral (Table 4).

**Figure 4:**
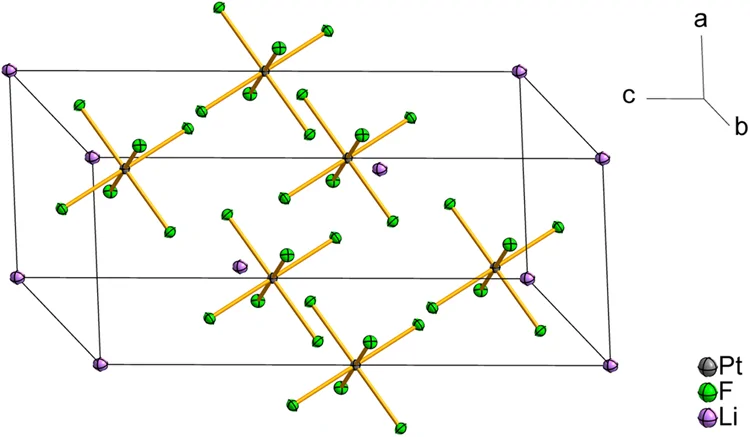
Section of the crystal structure of LiPtF_6_. Li–F bonds have been omitted for clarity. Displacement ellipsoids are shown at the 50% probability level.

In the KPtF_6_ and RbPtF_6_ structures, both of which adopt the KAsF_6_ structure type (Figure 5), each alkali-metal cation is coordinated by 12 F atoms in a slightly distorted cuboctahedron geometry comprising six shorter and six longer M–F bonds (KPtF_6_: 2.8369(14) and 2.9367(15) Å; RbPtF_6_: 2.9290(13) and 3.0600(13) Å) (Table 4). The 12 coordinating F atoms originate from eight [PtF_6_]^−^ anions, two of which coordinate the alkali-metal cation in a tridentate manner and six monodentately. These interactions produce a CsCl-type arrangement of the Pt and alkali-metal centers (Figure S8). The Pt–F bond lengths in KPtF_6_ (1.8855(13) Å) and in RbPtF_6_ (1.8843(11) Å) agree within 3σ with those in LiPtF_6_ and NaPtF_6_. The *cis*-F–Pt–F angles in the [PtF_6_]^−^ anions deviate slightly more from 90° than those in the lighter analogues (KPtF_6_: 88.07(7) and 91.93(7)°; RbPtF_6_: 88.61(6) and 91.39(6)°), whereas the *trans*-F–Pt–F angles remain exactly linear.

**Figure 5:**
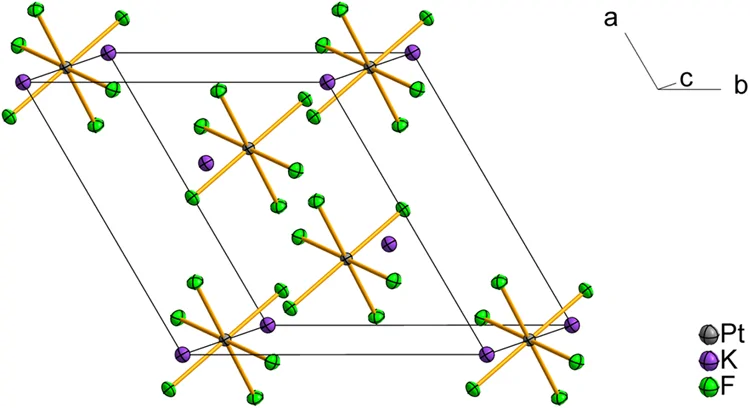
Section of the crystal structure of KPtF_6_. K–F bonds have been omitted for clarity. Displacement ellipsoids are shown at the 50% probability level.

### Raman Spectroscopy

In XeF_2_ coordination chemistry, Raman spectroscopy provides a rapid and straightforward means of establishing the presence of XeF_2_ ligation to metal centers. The intense symmetric Xe–F stretching band (497 cm^–1^)^39^ typically shifts to higher wavenumbers upon coordination. Its exact position depends on the Lewis acidity of the metal cation and whether XeF_2_ acts as a terminal or bridging ligand. For weakly Lewis-acidic monovalent centers, or for XeF_2_ molecules bridging two metal cations, the Xe–F stretching bands generally occur at lower wavenumbers (500–540 cm^–1^),^4,7–9^ whereas coordination to more strongly Lewis-acidic species, such as Mg^2+^ or Cu^2+^, can shift these bands to higher values (>575 cm^–1^).^12,14^ The Raman spectrum of [Li­(XeF_2_)_3_PtF_6_] (Figure 6, Table S16) exhibits a very strong, slightly broadened Xe–F stretching band (519 cm^–1^) of bridging XeF_2_ ligands, similar to those observed for other [Li­(XeF_2_)_*n*_AF_6_] (A = P, As, Ru, Ir) complexes.^9^ By contrast, the corresponding bands in the Raman spectrum of [Na­(XeF_2_)­PtF_6_] are markedly weaker and appear as four features (509–548 cm^–1^). Their comparatively low intensities likely reflect the low XeF_2_-to-metal-cation ratio. In the spectrum of [K­(XeF_2_)_2_PtF_6_], the Xe–F stretch again appears as a strong band (523 cm^–1^) with a shoulder (527 cm^–1^). These bands occur at appreciably higher values than the doublets observed for the related complexes [Ag­(XeF_2_)_2_AsF_6_] and [Ag­(XeF_2_)_2_PF_6_] (500–510 cm^–1^).^7,8^ Additional characteristic features in the spectra of [M­(XeF_2_)_*n*_PtF_6_] compounds arise from the Raman-active octahedral modes ν_1_, ν_2_, and ν_5_ of the [PtF_6_]^−^ anion. Owing to symmetry lowering and the distinct coordination environments of the fluorine atoms, several of these bands are split and occur within characteristic regions (ν_1_: 630–660 cm^–1^; ν_2_: 570–590 cm^–1^; ν_5_: 220–250 cm^–1^).

**Figure 6:**
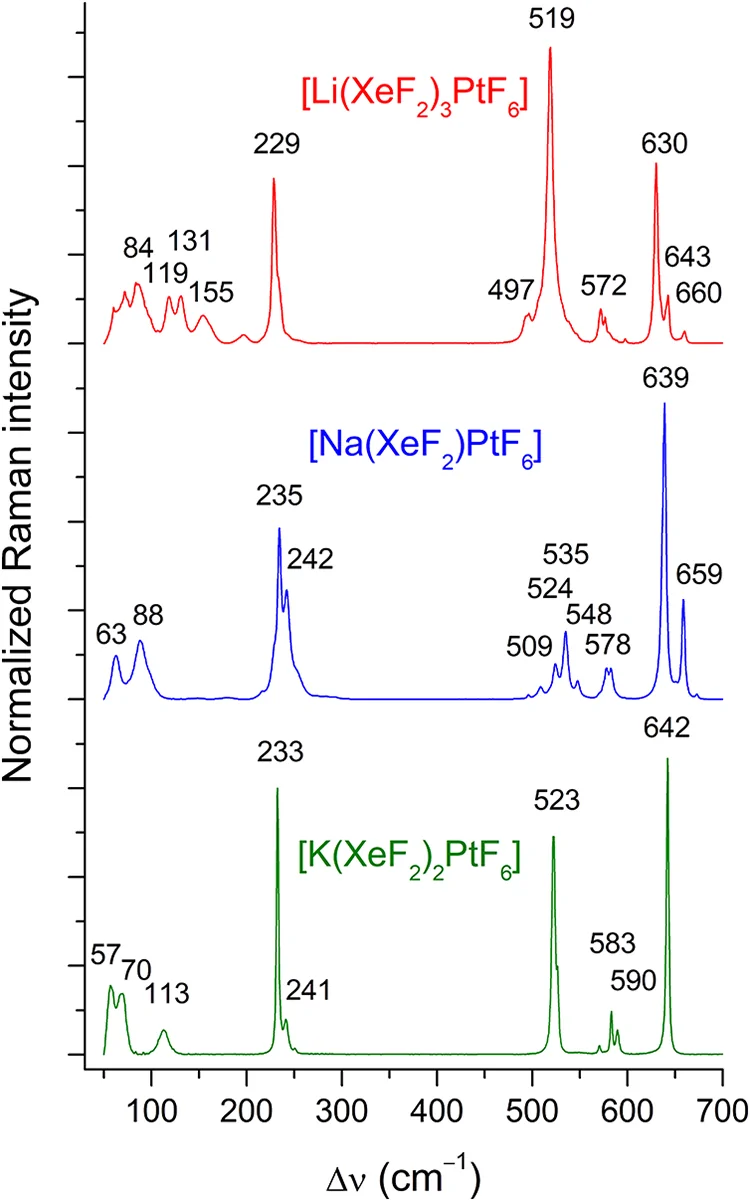
Low-temperature (100 K) Raman spectra of [Li­(XeF_2_)_3_PtF_6_] (red), [Na­(XeF_2_)­PtF_6_] (blue), and [K­(XeF_2_)_2_PtF_6_] (green).

Raman spectra of MPtF_6_ (M = Li, Na, K, Rb) compounds (Figure 7) exhibit all three Raman-active modes of the octahedral [PtF_6_]^−^ anion: ν_1_, ν_2_, and ν_5_. The ν_5_ band is split for LiPtF_6_, KPtF_6_, and RbPtF_6_, consistent with slight deviations from ideal octahedral symmetry. The ν_1_ band shifts systematically to lower wavenumbers with increasing atomic number of the alkali metal, from LiPtF_6_ (652 cm^–1^) to RbPtF_6_ (639 cm^–1^). This trend extends to the previously characterized CsPtF_6_ (636 cm^–1^),^38^ and an analogous trend has been reported for the alkali-metal hexafluoridomolybdates­(V).^40^

**Figure 7:**
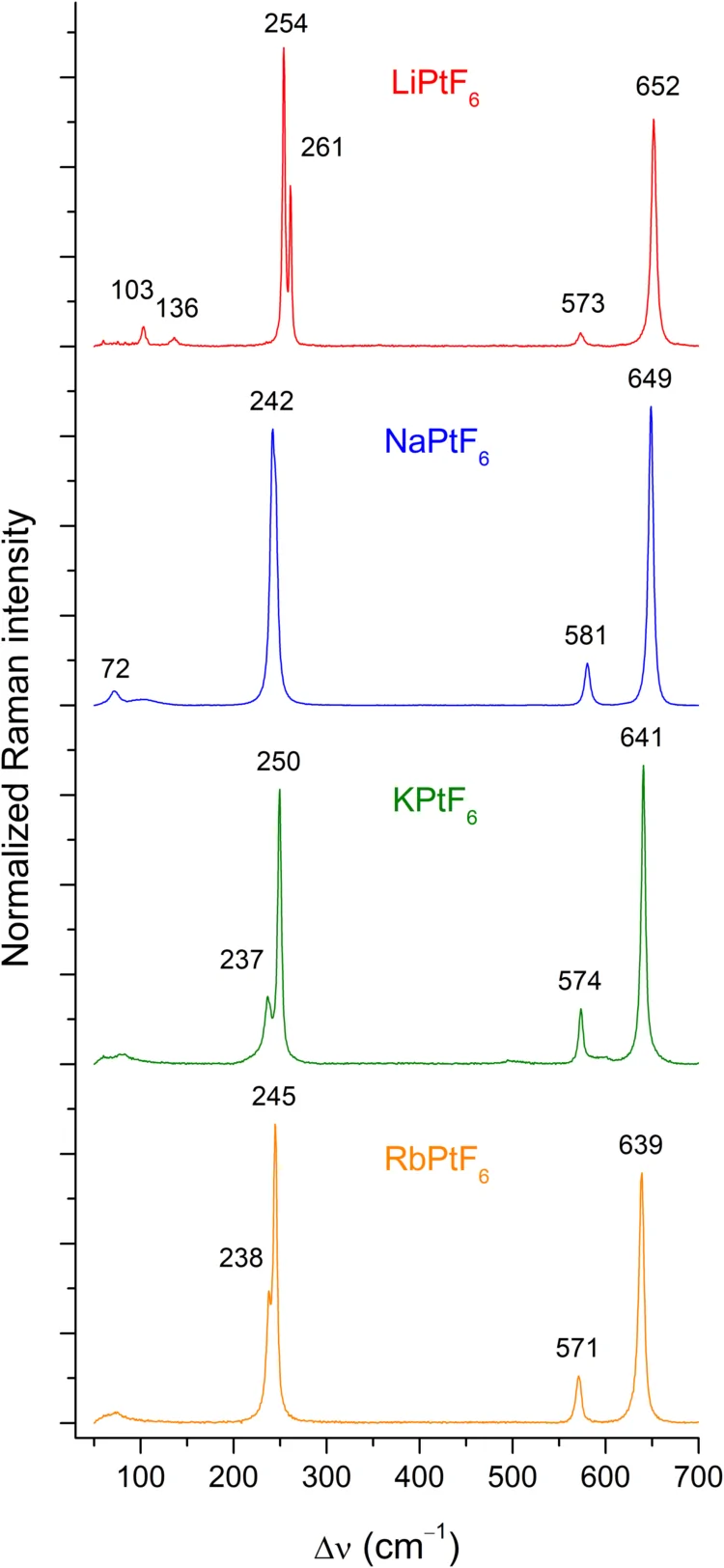
Ambient-temperature Raman spectra of LiPtF_6_ (red), NaPtF_6_ (blue), KPtF_6_ (green), and RbPtF_6_ (orange).

## Conclusions

In the present work, the ability of XeF_2_ to act as a ligand toward alkali-metal cations was reexamined through reactions with MPtF_6_ salts (M = Li, Na, K, Rb) in aHF. Slow crystallization by solvent evaporation from the resulting orange-yellow solutions at room temperature afforded single crystals of [Li­(XeF_2_)_3_PtF_6_], [Na­(XeF_2_)­PtF_6_], and [K­(XeF_2_)_2_PtF_6_] that were analyzed by SCXRD and Raman spectroscopy. These results contrast with earlier attempts to synthesize XeF_2_ coordination compounds of alkali metal cations in which only Li^+^ complexes were unambiguously identified and crystallization of [Li­(XeF_2_)_3_AsF_6_] required solvent removal at −50 °C. The present findings further demonstrate that the anion selection decisively influences both complex stability and the range of metal cations that can be ligated by XeF_2_. The presented complexes expand XeF_2_ coordination chemistry by providing the first example of XeF_2_ coordination to K^+^ and the first homoleptic XeF_2_ coordination sphere around a monovalent metal cation, [Li­(XeF_2_)_6_]^+^, in [Li­(XeF_2_)_3_PtF_6_]. Moreover, [Na­(XeF_2_)­PtF_6_] is a rare example of XeF_2_ coordination to Na^+^ and the first such complex containing a “classical” weakly coordinating anion AF_6_^–^. The single-crystal structures of the parent MPtF_6_ salts (M = Li, Na, K, Rb) were determined; these compounds had remained poorly characterized despite having been first reported more than six decades ago. Their Raman spectra are also reported and should facilitate the identification of these compounds in future studies.

## Experimental Section

*Caution: Handling of anhydrous HF (aHF), F*_2_ *, and high-valent fluorides requires great care and should be done only in a well-ventilated fume hood by properly trained personnel. It is imperative that protective equipment be worn at all times and that proper treatment procedures be available in the event of exposure*.^41–43^

### General Synthetic Procedures

A previously described vacuum line, constructed of nickel, copper, poly­(tetrafluoroethylene) (PTFE), and fluorinated ethylene propylene copolymer (FEP) was used for the work involving volatiles F_2_ and aHF (Figure S9).^44^ Solid materials were handled in a nitrogen atmosphere glovebox (Vigor SG1200/750E–SG1500/750E), with moisture content always kept below 1 ppm. Reactions were performed in h-shaped vessels, constructed from two FEP tubes (8 mm outside diameter and 6 mm inside diameter), one straight and one angled at 90°, both connected to a PTFE or poly­(chlorotrifluoroethylene) (PCTFE) valve via a brass-encased PTFE T-junction (Figure S10). Volume of the reactors was approximately 12.5 mL. Prior to their use, reaction vessels were passivated with 300–600 Torr of F_2_ overnight. The UV light was emitted by a 365 nm LED light source with an electrical power rating of 100 W.

### Reagents

F_2_ (Solvay Fluor, 98–99%), LiF (Aldrich, ≥99.98%), NaF (Alfa Aesar, 99.99%), KF (Acros Organics, 99.99%), RbF (Alfa Aesar, 99.975%) and Pt powder (Thermo Scientific, 99.98%) were used as supplied. Prior to use, the solvent aHF (Linde, 99.995%) was condensed in an FEP vessel loaded with K_2_NiF_6_ (Advance Research Chemicals, 99.9%) and stirred overnight to eliminate residual traces of moisture. XeF_2_ was synthesized via a gas-phase photochemical reaction between UV-photolyzed F_2_ and Xe, as previously described.^45^

Alkali-metal hexafluoridoplatinates­(V) were synthesized using the literature procedure for LiPtF_6_.^26^ Under inert atmosphere of a glovebox, alkali fluorides and Pt powder in a 1:1 molar ratio were weighed into the main arms of h-shaped FEP reactors, which also contained PTFE-coated stirring bars. The reactors were connected to the vacuum line where aHF and an approximately 2–3-fold excess of F_2_ were dosed into the reactors, which were then placed on a shaker and exposed to UV irradiation. After a day, the reactors were reconnected to the vacuum line and frozen in liquid nitrogen, evacuating all volatiles at that temperature before recharging them with F_2_ and placing them again, with agitation, under UV light. After a period of 22 h, no visible changes in the contents could be observed and the yellow solids were isolated by removing all volatiles from the reactors at room temperature.

### Synthesis and Crystal Growth of [Li­(XeF_2_)_3_PtF_6_]

XeF_2_ (230 mg, 1.359 mmol) was added to the main arm of an h-shaped FEP vessel containing LiPtF_6_, which had been synthesized from LiF (10 mg, 0.386 mmol) and Pt (75 mg, 0.384 mmol). aHF (1.2 mL) was condensed to the vessel, forming an orange-yellow solution with no visible precipitate. The solution was decanted into the side arm of the vessel. The main arm was then placed in a cooling bath thermostat containing EtOH, while the side arm containing the solution remained at room temperature. Over the next 4 days, the temperature difference between the two arms was gradually increased from 8 to 43 °C by lowering the temperature of the cooling bath. Consequently, aHF evaporated from the side arm and condensed in the main arm, resulting in the formation of yellow crystals in the side arm. The product was isolated by removing the volatiles from the vessel while keeping the contents of the side arm cooled in an EtOH bath maintained below −25 °C.

### Synthesis and Crystal Growth of [Na­(XeF_2_)­PtF_6_]

XeF_2_ (125 mg, 0.738 mmol) was added to the main arm of the vessel, which also contained NaPtF_6_, prepared from NaF (9 mg, 0.214 mmol) and Pt (41 mg, 0.210 mmol). aHF (1.2 mL) was introduced, affording a bright orange-yellow solution with no visible precipitate. After stirring for 30 min, the solution was decanted into the side arm, and the main arm was placed in a cooling bath thermostat. Over the next 4 days, the temperature difference between the two arms was gradually increased from 4 to 48 °C. A yellow-orange solid containing crystals of the sodium complex remained in the side arm. The product was isolated by removing the volatiles from the vessel while maintaining the side arm at a temperature below −25 °C.

### Synthesis and Crystal Growth of [K­(XeF_2_)_2_PtF_6_]

XeF_2_ (84 mg, 0.496 mmol) was added to the main arm of a reactor containing KPtF_6_, synthesized from KF (9 mg, 0.155 mmol) and Pt (30 mg, 0.154 mmol). aHF (1 mL) was condensed into the reactor, forming a vibrant orange-yellow solution with a small amount of insoluble yellow precipitate. In an attempt to dissolve the precipitate, the reaction mixture was stirred overnight. However, by the following morning, approximately the same amount of yellow solid persisted and had consolidated into a single chunk. Stirring was then halted, and the clear supernatant was decanted into the side arm of the reactor and the main arm was placed in a cooling-bath thermostat. When the temperature difference between the two arms exceeded 18 °C, large cubic yellow crystals began to form. The product was isolated by removing the volatiles while maintaining the side arm below −15 °C.

### Attempted Synthesis of [Rb­(XeF_2_)_*n*_PtF_6_]

XeF_2_ (80 mg, 0.473 mmol) was added to the main arm, which contained RbPtF_6_, synthesized from RbF (16 mg, 0.153 mmol) and Pt (30 mg, 0.154 mmol). aHF (1 mL) solvent was condensed to the reactor, forming a bright orange-yellow solution with insoluble yellow precipitate remaining at the bottom of the reactor, which persisted even after 24 h of stirring. The clear yellow supernatant was decanted into the side arm of the reactor and the main arm was then placed into a cooling bath thermostat. As the temperature difference between the two arms was increased above 7 °C, the formation of small yellow crystals was observed. The crystals of RbPtF_6_ were isolated by removing the volatiles from the reactor while keeping the contents of the side arm cooled at temperatures below −20 °C.

### Crystal Mounting

The crystalline samples were placed on a watch glass and covered with inert perfluorodecalin oil (Fluorochem, 96.0%) in an inert-atmosphere glovebox. Outside the glovebox, suitable crystals were selected under polarized light of a stereomicroscope and mounted on a MiTeGen dual-thickness microloop using Baysilone paste (Bayer-Silicone, mittelviskos). Immediately after the pin assembly was lifted above the surface of the perfluorodecalin, it was grabbed with cryo pin tongs cooled to −196 °C and rapidly transferred to the goniometer head of the diffractometer, where the crystal was protected by a stream of cold nitrogen gas.

### Single-Crystal X-ray Data Collection and Reduction

Single-crystal X-ray diffraction data were collected using microfocused Ag Kα radiation (λ = 0.56087 Å) on a Rigaku OD XtaLAB Synergy-S diffractometer equipped with a Dectris EIGER2 R CdTe 1M hybrid pixel-array detector and an Oxford Cryosystems 800 Series Cryostream (100 K). *CrysAlisPro* was employed for data processing, including empirical and numerical absorption correction, as well as to identify and integrate two twin domains in [Na­(XeF_2_)­PtF_6_], and to generate the corresponding .hkl4 and .hkl5 files.^46^ The crystal structures of XeF_2_ coordination compounds were solved using charge flipping with *SUPERFLIP*,^47^ whereas the crystal structures of alkali hexafluoridoplatinates were solved using the Patterson method implemented in *SHELXS*-2016.^48^ All structures were refined with *SHELXL* ^49^ within *Olex2* software.^50^ During refinement, the refined twin-domain ratio for [Na­(XeF_2_)­PtF_6_] converged to 0.8983(6):0.1017(6). The crystal structure of [Li­(XeF_2_)_3_PtF_6_] was modeled as an inversion twin, with refined twin fractions of 0.976(3) and 0.024(3). Figures were generated using *DIAMOND* software.^51^

### Computational Details

Periodic DFT calculations were performed in CASTEP (v. 26.1.1)^52^ using the generalized gradient approximation (GGA) PBEsol functional^53^ with the Tkatchenko–Scheffler (TS) van der Waals correction scheme,^54^ and the meta-GGA rSCAN functional^55^ with the D4 dispersion correction.^56^ Norm-conserving pseudopotentials were employed with a plane-wave kinetic-energy cutoff of 1100 eV and an electronic energy convergence threshold of 10^–7^ eV. Brillouin-zone integrations were performed using Monkhorst–Pack *k*-point meshes with a spacing of 2π × 0.04 Å^–1^. Crystal structures were optimized (Tables S17–S23) with respect to all free structural parameters, i.e., all parameters not constrained by the space-group symmetry. The geometry optimization convergence criteria were 0.02 eV Å^–1^ for atomic forces, 0.05 GPa for stresses, and 5 × 10^–4^ Å for atomic displacements. Atomic charges were calculated from the ground-state electron density using the Hirshfeld partitioning scheme.^57^

### Raman Spectroscopy

Raman spectra were acquired on samples contained in glass capillaries prepared as previously described^44^ using a Bruker Senterra II confocal Raman microscope over the range 50–1410 cm^–1^ with a spectral resolution of 1.5 cm^–1^ and an aperture of 15 μm × 1000 μm. A 785 nm laser with a nominal power output of 100 mW was used for excitation. Low-temperature (100 K) measurements were performed using a Linkam LTS420 low-temperature stage. Raman spectra of the MPtF_6_ compounds were acquired on powdered samples, whereas the spectra of the [M­(XeF_2_)_*n*_PtF_6_] complexes were recorded on several randomly oriented single crystals and subsequently averaged. The averaged spectra comprised seven individual spectra for Li, nine for Na, and six for K complex. Background subtraction was performed on all spectra using three iterations of the concave rubber-band correction with 64 baseline points. Background subtraction and averaging were performed using Bruker *Opus* 8.7 software suite.

## Supplementary Material



## Data Availability

The experimental data supporting the findings of this study are available in the Zenodo open repository (https://zenodo.org/) under the DOI number: 10.5281/zenodo.22259609.

## Notes

Crystallographic data, crystal packing, tabulated Raman data; atomic coordinates of the DFT-optimized structures; and photographs of the vacuum line and the h-shaped FEP vessel (PDF)

## ∥

Faculty of Chemistry and Chemical Engineering, University of Maribor, Smetanova ulica 17, 2000 Maribor, Slovenia

## Notes

Dedicated to Professor Emeritus Rika Hagiwara on the 35th anniversary of the first report of a complex containing a noble-gas fluoride ligand coordinated to a "naked" metal cation, [Ag­(XeF_2_)_2_AsF_6_].
