# Supplementary material for: Alkali-Metal Complexes of XeF2: Synthesis and Structural Characterization of [Li(XeF2)3PtF6], [Na(XeF2)PtF6], and [K(XeF2)2PtF6]
Source: Inorg Chem. 2026 Oct 11;65(42):24961–9. doi: 10.1021/acs.inorgchem.6c04065 (PMC13722038; doi:10.1021/acs.inorgchem.6c04065)
Supplement: Supplementary file 1 [file ic6c04065_si_001.pdf]

Supporting Information

# Alkali-Metal Complexes of $XeF_2$: Synthesis and Structural Characterization of $[Li(XeF_2)_3PtF_6]$, $[Na(XeF_2)PtF_6]$, and $[K(XeF_2)_2PtF_6]$

*Klemen Motaln,[1,2,†] Dominik Kurzydłowski,[3] Matic Lozinšek[1,2,]**

*[1] Extreme Conditions Chemistry Laboratory, Jožef Stefan Institute, Jamova cesta 39, 1000 Ljubljana, Slovenia*

*[2] Jožef Stefan International Postgraduate School, Jamova cesta 39, 1000 Ljubljana, Slovenia*

*[3] Faculty of Mathematics and Natural Sciences, Cardinal Stefan Wyszyński University, ul. Wóycickiego 1/3, 01-938 Warsaw, Poland*

*[†] Present address: Faculty of Chemistry and Chemical Engineering, University of Maribor, Smetanova ulica 17, 2000 Maribor, Slovenia*

**Corresponding author's e-mail: matic.lozinsek@ijs.si*

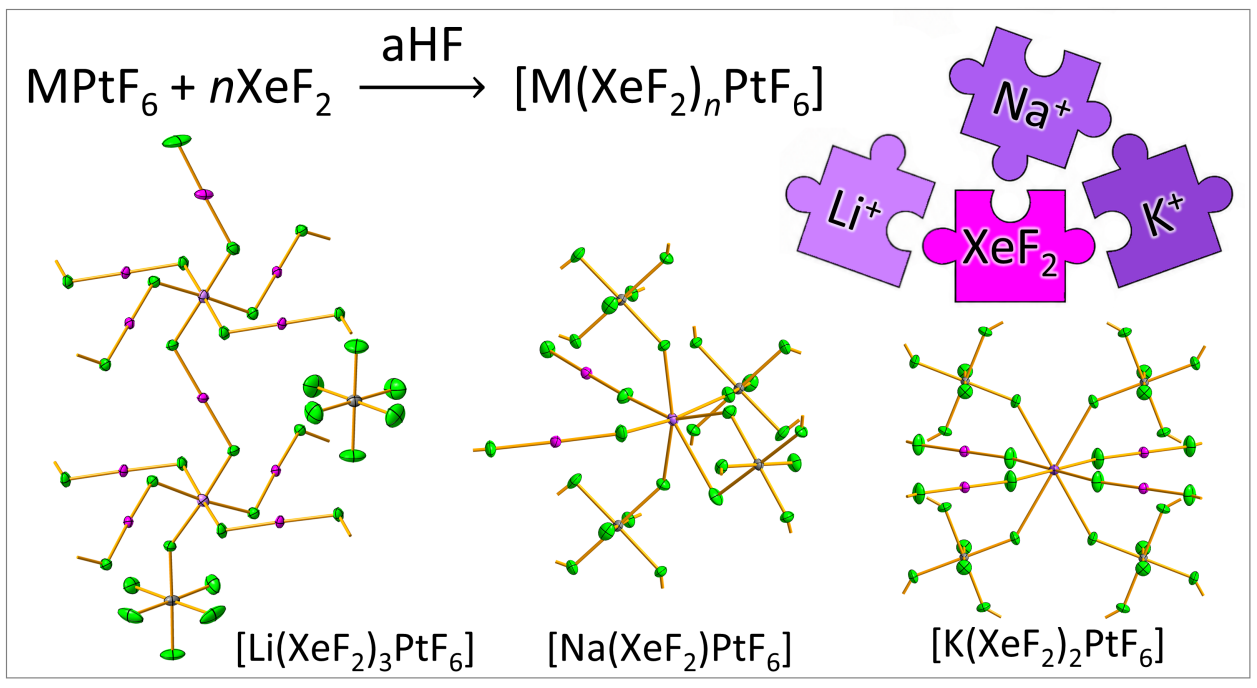


DOI: 10.1021/acs.inorgchem.6c04065

# Table of Contents

**Table S1**. Crystallographic data and refinement results for [$Li(XeF_2)_3PtF_6$], [$Na(XeF_2)PtF_6$], and [$K(XeF_2)_2PtF_6$]. ........ 4

**Table S2.** Experimental (SCXRD) and calculated (PBEsol and rSCAN) bond lengths in [$Li(XeF_2)_3PtF_6$], the corresponding differences, and calculated bond valences (BV) and bond-valence sums for $Li^+$. ........ 5

**Figure S1.** Crystal packing of [$Li(XeF_2)_3PtF_6$] viewed along the *a* axis, illustrating the double-layer structure, with the layers lying parallel to the *ac* crystallographic plane. ........ 7

**Table S3.** Experimental (SCXRD) and calculated (PBEsol and rSCAN) bond lengths in [$Na(XeF_2)PtF_6$], the corresponding differences, and calculated bond valences (BV) and bond-valence sum for $Na^+$. ........ 8

**Figure S2.** Section of the crystal structure of [$Na(XeF_2)PtF_6$] showing a single layer parallel to the *bc* plane, highlighting the bridging role of the $XeF_2$ ligands (top), and the stacking of such layers along the *a* axis to generate a three-dimensional structure (bottom). ........ 9

**Table S4.** Experimental (SCXRD) and calculated (PBEsol and rSCAN) bond lengths in [$K(XeF_2)_2PtF_6$], the corresponding differences, and calculated bond valences (BV) and bond-valence sum for $K^+$. ........ 10

**Figure S3.** Alternative view of the eight-coordinate $K^+$ cation in the crystal structure of [$K(XeF_2)_2PtF_6$], highlighting its distorted cube-like coordination geometry. ........ 11

**Figure S4.** Section of the crystal structure of [$K(XeF_2)_2PtF_6$] showing a layer composed of [$PtF_6$] and [$KF_8$] units ($XeF_2$ ligands omitted for clarity) lying parallel to the *ab* plane (top), and the interconnection of these layers into a three-dimensional structure by bridging $XeF_2$ ligands (bottom). ........ 12

**Table S5.** Hirshfeld atomic charges (e) in [$Li(XeF_2)_3PtF_6$], calculated at the PBEsol-TS and rSCAN-D4 levels of theory. ........ 13

**Table S6.** Hirshfeld atomic charges (e) in [$Na(XeF_2)PtF_6$], calculated at the PBEsol-TS and rSCAN-D4 levels of theory. ........ 14

**Table S7.** Hirshfeld atomic charges (e) in [$K(XeF_2)_2PtF_6$], calculated at the PBEsol-TS and rSCAN-D4 levels of theory. ........ 14

**Table S8.** Calculated energy changes ($\Delta E$) for the formation of the three $XeF_2$ coordination compounds calculated at the PBEsol-TS and the rSCAN-D4 levels of theory, expressed in both eV and kJ per mol of the product. ........ 15

**Figure S5.** Plot of the energy changes ($\Delta E$) calculated at the PBEsol-TS and rSCAN-D4 levels of theory for the formation of the three coordination compounds. ........ 15

**Table S9**. Crystallographic data and refinement results for $LiPtF_6$, $NaPtF_6$, $KPtF_6$, and $RbPtF_6$. ........ 16

**Table S10.** Li−F bond lengths and bond-valence calculations for $Li^+$ in $LiPtF_6$. ........ 17

**Table S11.** Na−F bond lengths and bond-valence calculations for $Na^+$ in $NaPtF_6$. ........ 17

**Table S12.** K−F bond lengths and bond-valence calculations for $K^+$ in $KPtF_6$. ........ 18

**Table S13.** Rb−F bond lengths and bond-valence calculations for $Rb^+$ in $RbPtF_6$. ........ 19

**Table S14.** Calculated energies of the $MPtF_6$ (M = Li, Na, K, Rb) compounds in the $LiSbF_6$- and $KAsF_6$-type structures calculated at the PBEsol-TS and rSCAN-D4 levels of theory. ........ 20

**Table S15.** Calculated unit-cell volumes of the $MPtF_6$ (M = Li, Na, K, Rb) compounds in the $LiSbF_6$- and $KAsF_6$-type structures calculated at the PBEsol-TS and rSCAN-D4 levels of theory ............................... 20

**Figure S6.** Plot comparing the unit-cell volumes calculated for $MPtF_6$ (M = Li, Na, K, Rb) in the $LiSbF_6$ and $KAsF_6$ structure types at the PBEsol-TS and rSCAN-D4 levels of theory. ........................................ 20

**Figure S7.** Coordination environment of the $Na^+$ cation, illustrating a slightly distorted octahedral geometry (top), and the crystal packing of $NaPtF_6$ viewed along the *a* crystallographic axis (bottom). An analogous arrangement is observed in the crystal structure of $LiPtF_6$. ............................................ 21

**Figure S8.** Coordination environment of the $Rb^+$ cation, illustrating a distorted cuboctahedron geometry (top), and the crystal packing of $RbPtF_6$ viewed along the *c* crystallographic axis (bottom). An analogous arrangement is observed in the crystal structure of $KPtF_6$. ............................................ 22

**Table S16.** Observed bands in the low-temperature Raman spectra of $[Li(XeF_2)_3PtF_6]$, $[Na(XeF_2)PtF_6]$, and $[K(XeF_2)_2PtF_6]$ (Figure 6) and in the room-temperature Raman spectra of powdered samples of $LiPtF_6$, $NaPtF_6$, $KPtF_6$, and $RbPtF_6$ (Figure 7), with relative intensities given in parentheses. ................ 23

**Figure S9.** Vacuum line constructed from nickel, copper, polytetrafluoroethylene (PTFE), and fluorinated ethylene propylene (FEP) copolymer, used for the addition or removal of volatile species. ........................................................................................................................................ 24

**Figure S10.** The h-shaped vessel, constructed from FEP tubing and brass-encased PTFE components, was used for the reactions described in this study. Throughout the manuscript, the angled arm visible on the left is termed the side arm, whereas the straight arm extending vertically from the T-junction is termed the main arm. ........................................................................................................ 24

**Table S17.** Atomic coordinates of the DFT-optimized crystal structure of $[Li(XeF_2)_3PtF_6]$ .................... 25

**Table S18.** Atomic coordinates of the DFT-optimized crystal structure of $[Na(XeF_2)PtF_6]$ .................... 27

**Table S19.** Atomic coordinates of the DFT-optimized crystal structure of $[K(XeF_2)_2PtF_6]$ ..................... 28

**Table S20.** Atomic coordinates of the DFT-optimized crystal structure of $LiPtF_6$ ................................. 28

**Table S21.** Atomic coordinates of the DFT-optimized crystal structure of $NaPtF_6$ ............................... 29

**Table S22.** Atomic coordinates of the DFT-optimized crystal structure of $KPtF_6$ .................................. 29

**Table S23.** Atomic coordinates of the DFT-optimized crystal structure of $RbPtF_6$ ............................... 30

**References** ......................................................................................................................... 31

**Table S1**. Crystallographic data and refinement results for [Li($XeF_2$)$_3$$PtF_6$], [Na($XeF_2$)$PtF_6$], and [K($XeF_2$)$_2$$PtF_6$].

| Compound | [Li($XeF_2$)$_3$$PtF_6$] | [Na($XeF_2$)$PtF_6$] | [K($XeF_2$)$_2$$PtF_6$] |
|---|---|---|---|
| Formula | $Xe_3PtLiF_{12}$ | $XePtNaF_8$ | $Xe_2PtKF_{10}$ |
| $F_w$ | 823.93 | 501.38 | 686.79 |
| $T$ (K) | 100.00(10) | 100.00(10) | 100.00(10) |
| Crystal system | Monoclinic | Monoclinic | Tetragonal |
| Space group | $P2_1$ | $P2_1/c$ | $I4/mcm$ |
| $a$ (Å) | 6.80526(6) | 7.1459(3) | 8.73777(9) |
| $b$ (Å) | 26.79621(19) | 10.0427(4) | 8.73777(9) |
| $c$ (Å) | 6.81133(6) | 10.2322(4) | 13.3131(2) |
| α (°) | 90 | 90 | 90 |
| β (°) | 92.6673(8) | 95.852(4) | 90 |
| γ (°) | 90 | 90 | 90 |
| $V$ (Å$^3$) | 1240.736(17) | 730.47(5) | 1016.44(3) |
| $Z$ | 4 | 4 | 4 |
| $\rho_{calc}$ (g/cm$^3$) | 4.411 | 4.559 | 4.488 |
| Crystal size (mm) | 0.164 × 0.159 × 0.13 | 0.111 × 0.086 × 0.085 | 0.188 × 0.128 × 0.114 |
| Radiation type | Ag *K*α | Ag *K*α | Ag *K*α |
| λ (Å) | 0.56087 | 0.56087 | 0.56087 |
| μ (mm$^{-1}$) | 10.363 | 12.918 | 11.227 |
| $F$(000) | 1404 | 860 | 1180 |
| $\theta_{max}$ (°) | 36.3860 | 36.5000 | 36.8940 |
| Index ranges | $-14 \le h \le 14$ | $-15 \le h \le 14$ | $-18 \le h \le 18$ |
| | $-56 \le k \le 57$ | $-21 \le k \le 21$ | $-18 \le k \le 18$ |
| | $-14 \le l \le 14$ | $-21 \le l \le 21$ | $-27 \le l \le 27$ |
| Reflections collected | 222318 | 9650 | 45162 |
| Independent reflections | 24060 | 9650 | 1396 |
| Reflections with [$I > 2\sigma(I)$] | 21733 | 8612 | 1023 |
| $R_{int}$ | 0.0799 | - | 0.0810 |
| $R_{sigma}$ | 0.0314 | 0.0236 | 0.0174 |
| Data/restraints/parameters | 24060/1/309 | 9650/0/101 | 1396/0/26 |
| $S$ | 1.034 | 1.051 | 1.101 |
| $R_1$, $wR_2$ [$I > 2\sigma(I)$] | 0.0284, 0.0686 | 0.0358, 0.0996 | 0.0174, 0.0458 |
| $R_1$, $wR_2$ [all data] | 0.0330, 0.0706 | 0.0413, 0.1042 | 0.0251, 0.0495 |
| $\Delta\rho_{min}$, $\Delta\rho_{max}$ (e/Å$^3$) | −3.231, 3.445 | −3.226, 3.440 | −1.576, 1.525 |
| Absolute structure | Refined as an inversion twin. | - | - |

**Table S2.** Experimental (SCXRD) and calculated (PBEsol and rSCAN) bond lengths in [Li($XeF_2$)$_3$$PtF_6$], the corresponding differences, and calculated bond valences (BV) and bond-valence sums for $Li^+$.

| Bond | Experimental (Å) | BV (vu)[a] | PBEsol-TS (Å) | Δ (%)[b] | rSCAN-D4 (Å) | Δ (%)[b] |
|---|---|---|---|---|---|---|
| Xe1–F1 | 1.967(4) | | 2.009 | 2.1 | 2.004 | 1.9 |
| Xe1–F2 | 2.020(3) | | 2.061 | 2.0 | 2.055 | 1.7 |
| Xe2–F3 | 1.994(2) | | 2.033 | 2.0 | 2.026 | 1.6 |
| Xe2–F4 | 2.007(2) | | 2.042 | 1.7 | 2.037 | 1.5 |
| Xe3–F5 | 1.999(2) | | 2.035 | 1.8 | 2.029 | 1.5 |
| Xe3–F6 | 1.999(2) | | 2.040 | 2.1 | 2.033 | 1.7 |
| Xe4–F7 | 1.999(2) | | 2.034 | 1.8 | 2.027 | 1.4 |
| Xe4–F8 | 2.003(2) | | 2.039 | 1.8 | 2.033 | 1.5 |
| Xe5–F9 | 1.999(3) | | 2.038 | 2.0 | 2.032 | 1.7 |
| Xe5–F10 | 2.002(2) | | 2.036 | 1.7 | 2.030 | 1.4 |
| Xe6–F11 | 2.000(2) | | 2.041 | 2.1 | 2.029 | 1.5 |
| Xe6–F12 | 2.003(2) | | 2.035 | 1.6 | 2.034 | 1.5 |
| Pt1–F13 | 1.872(3) | | 1.897 | 1.3 | 1.886 | 0.7 |
| Pt1–F14 | 1.877(3) | | 1.894 | 0.9 | 1.883 | 0.3 |
| Pt1–F15 | 1.878(4) | | 1.892 | 0.7 | 1.901 | 1.2 |
| Pt1–F16 | 1.879(3) | | 1.892 | 0.7 | 1.882 | 0.2 |
| Pt1–F17 | 1.884(3) | | 1.896 | 0.6 | 1.885 | 0.1 |
| Pt1–F18 | 1.909(3) | | 1.933 | 1.3 | 1.939 | 1.6 |
| Pt2–F19 | 1.868(4) | | 1.892 | 1.3 | 1.885 | 0.9 |
| Pt2–F20 | 1.870(4) | | 1.896 | 1.4 | 1.894 | 1.3 |
| Pt2–F21 | 1.873(4) | | 1.897 | 1.3 | 1.894 | 1.1 |
| Pt2–F22 | 1.873(4) | | 1.896 | 1.2 | 1.893 | 1.1 |
| Pt2–F23 | 1.876(4) | | 1.896 | 1.1 | 1.893 | 0.9 |
| Pt2–F24 | 1.889(4) | | 1.897 | 0.4 | 1.891 | 0.1 |
| Li1–F3 | 2.032(8) | 0.163 | 2.030 | −0.1 | 1.971 | −3.0 |
| $Li1^{i}$–F4 | 1.953(8) | 0.201 | 1.955 | 0.1 | 1.909 | −2.4 |
| Li1–F7 | 1.962(8) | 0.197 | 1.958 | −0.2 | 1.915 | −2.4 |
| $Li1^{iii}$–F8 | 1.987(8) | 0.184 | 1.990 | 0.2 | 1.939 | −2.2 |

**Table S2.** Continued.

| Bond distance | Experimental (Å) | BV (vu)[a] | PBEsol-TS (Å) | Δ (%)[b] | rSCAN-D4 (Å) | Δ (%)[b] |
|---|---|---|---|---|---|---|
| Li1–F18 | 1.927(8) | 0.216 | 1.910 | −0.9 | 1.865 | −3.2 |
| $Li1^{iv}$–F10 | 2.011(8) | 0.172 | 2.025 | 0.7 | 1.967 | −2.2 |
| | SUM | **1.132** | | | | |
| Li2–F2 | 1.943(9) | 0.207 | 1.937 | −0.3 | 1.909 | −1.7 |
| Li2–F5 | 1.951(8) | 0.202 | 1.952 | 0.1 | 1.909 | −2.2 |
| $Li2^{ii}$–F6 | 1.986(8) | 0.184 | 1.974 | −0.6 | 1.919 | −3.4 |
| Li2–F9 | 1.994(9) | 0.180 | 1.993 | −0.1 | 1.938 | −2.8 |
| $Li2^{v}$–F11 | 1.966(7) | 0.194 | 1.961 | −0.3 | 1.915 | −2.6 |
| Li2–F12 | 1.976(7) | 0.189 | 1.970 | −0.3 | 1.919 | −2.9 |
| | SUM | **1.157** | | | | |

[a] Bond-valence parameters[1]: $R_0$ = 1.360 Å, $B$ = 0.37 Å

[b] Calculated as: $\Delta = ((d_{calc} - d_{exp})/d_{exp}) \times 100$

Symmetry codes: (i) $x - 1, y, z$; (ii) $x + 1, y, z$; (iii) $x, y, z - 1$; (iv) $-x + 1, y + 1/2, -z + 1$; (v) $x, y, z + 1$.

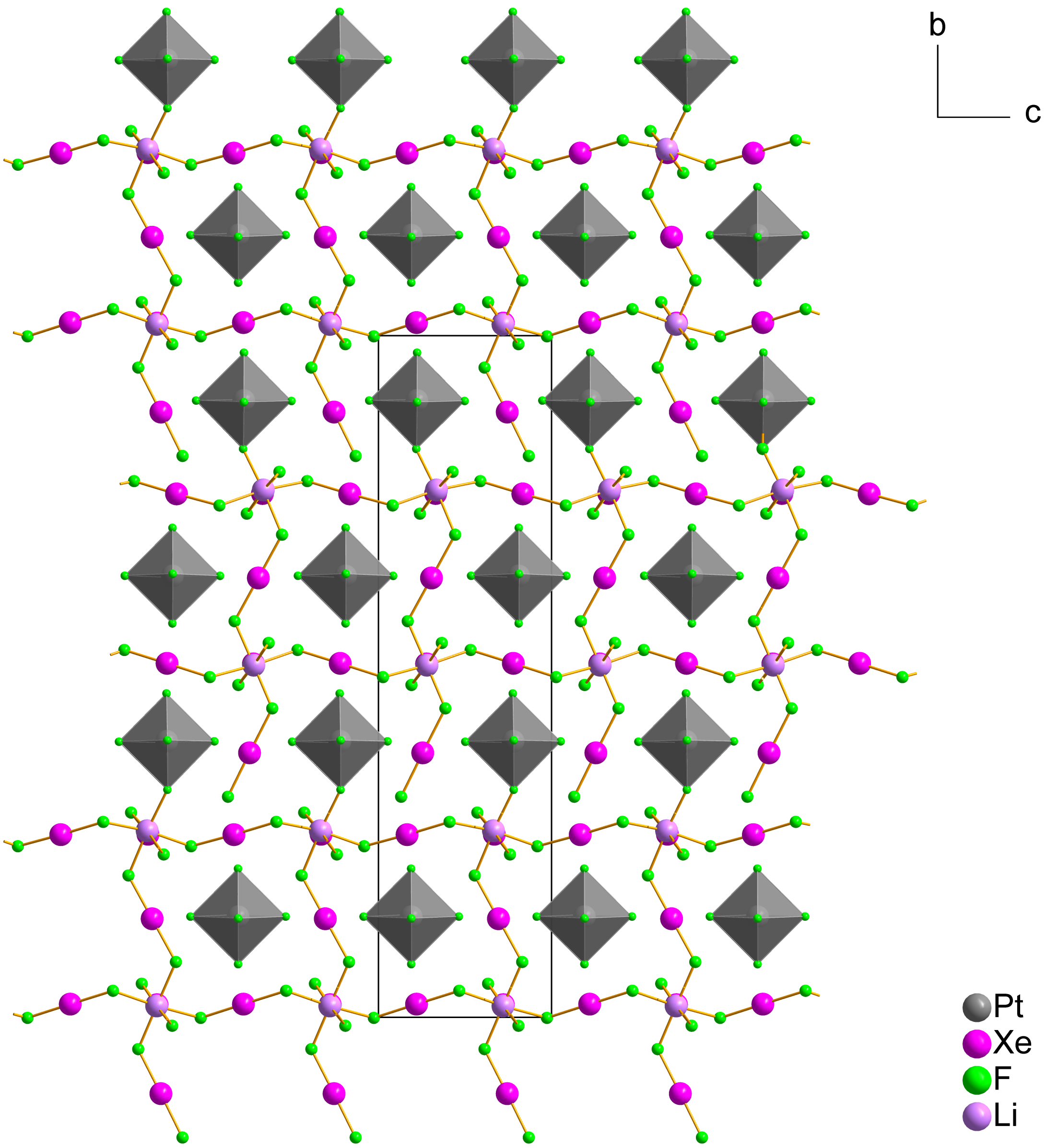


**Figure S1.** Crystal packing of [Li($XeF_2$)$_3$$PtF_6$] viewed along the *a* axis, illustrating the double-layer structure, with the layers lying parallel to the *ac* crystallographic plane.

**Table S3.** Experimental (SCXRD) and calculated (PBEsol and rSCAN) bond lengths in [Na($XeF_2$)$PtF_6$], the corresponding differences, and calculated bond valences (BV) and bond-valence sum for $Na^+$.

| Bond | Experimental (Å) | BV (vu)[a] | PBEsol-TS (Å) | Δ (%)[b] | rSCAN-D4 (Å) | Δ (%)[b] |
|---|---|---|---|---|---|---|
| Xe1−F1 | 1.977(2) | | 2.016 | 2.0 | 2.011 | 1.7 |
| Xe1−F2 | 1.997(2) | | 2.031 | 1.7 | 2.028 | 1.6 |
| Pt1−F3 | 1.864(2) | | 1.878 | 0.8 | 1.868 | 0.2 |
| Pt1−F4 | 1.880(2) | | 1.890 | 0.5 | 1.874 | −0.3 |
| Pt1−F5 | 1.884(2) | | 1.904 | 1.1 | 1.912 | 1.5 |
| Pt1−F6 | 1.891(2) | | 1.896 | 0.3 | 1.884 | −0.4 |
| Pt1−F7 | 1.891(2) | | 1.903 | 0.6 | 1.887 | −0.2 |
| Pt1−F8 | 1.897(2) | | 1.911 | 0.7 | 1.922 | 1.3 |
| Na1−F1$^{i}$ | 2.254(3) | 0.210 | 2.269 | 0.7 | 2.230 | −1.1 |
| Na1−F2 | 2.250(3) | 0.213 | 2.274 | 1.1 | 2.239 | −0.5 |
| Na1−F4 | 2.910(3) | 0.036 | 2.860 | −1.7 | 2.830 | −2.7 |
| Na1−F5$^{ii}$ | 2.299(3) | 0.186 | 2.281 | −0.8 | 2.232 | −2.9 |
| Na1−F6$^{iii}$ | 2.292(3) | 0.190 | 2.303 | 0.5 | 2.286 | −0.3 |
| Na1−F7$^{iv}$ | 2.365(3) | 0.156 | 2.403 | 1.6 | 2.379 | 0.6 |
| Na1−F8 | 2.336(3) | 0.168 | 2.303 | −1.4 | 2.272 | −2.7 |
| | **SUM** | **1.159** | | | | |

[a] Bond-valence parameters[1]: $R_0$ = 1.677 Å, $B$ = 0.37 Å

[b] Calculated as: $\Delta = ((d_{calc} - d_{exp})/d_{exp}) \times 100$

Symmetry codes: (i) $x$, $-y + 1/2$, $z + 1/2$; (ii) $-x + 1$, $y - 1/2$, $-z + 3/2$; (iii) $x$, $-y + 3/2$, $z + 1/2$; (iv) $-x$, $y - 1/2$, $-z + 3/2$.

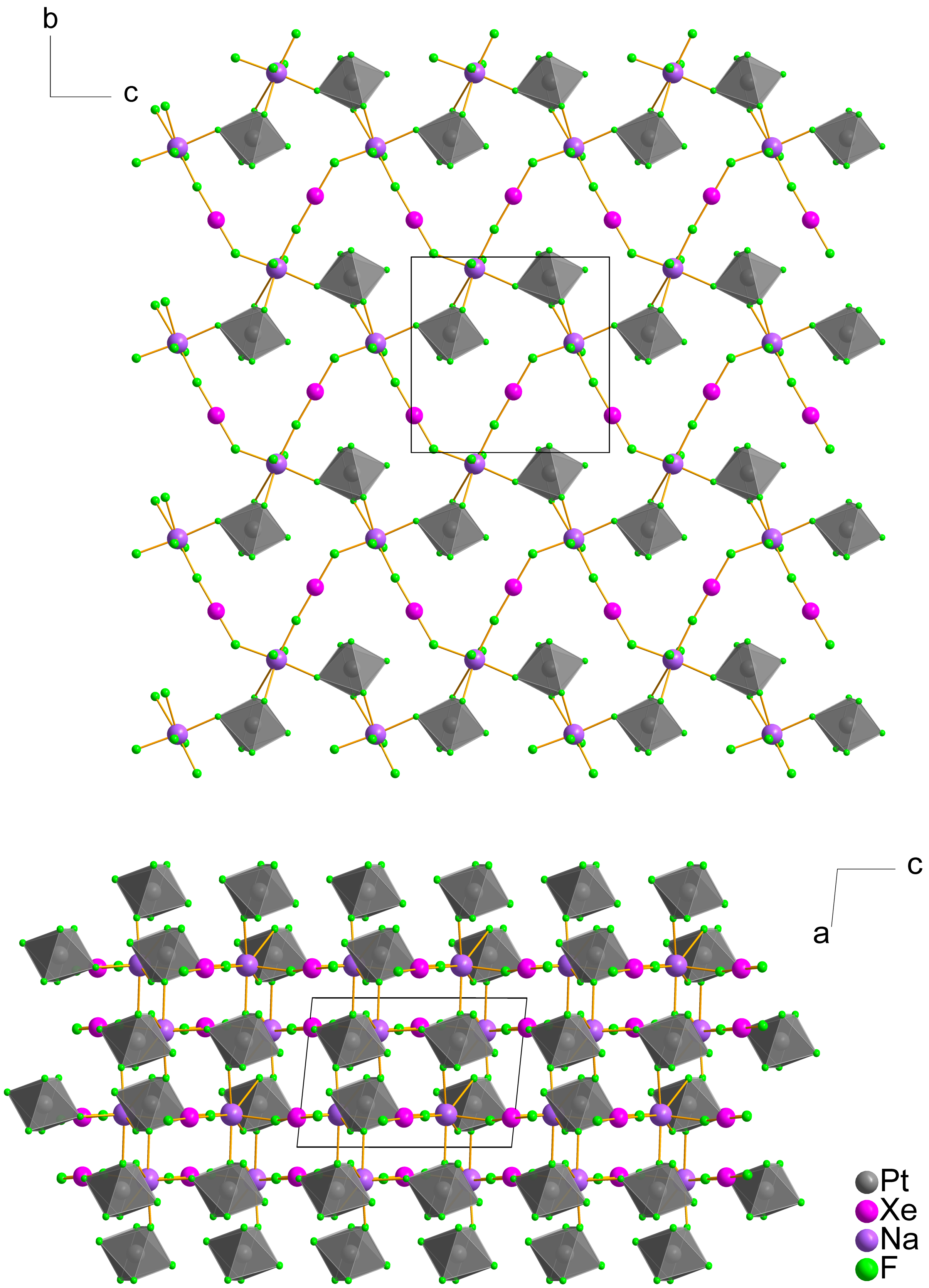


**Figure S2.** Section of the crystal structure of [$Na(XeF_2)PtF_6$] showing a single layer parallel to the *bc* plane, highlighting the bridging role of the $XeF_2$ ligands (top), and the stacking of such layers along the *a* axis to generate a three-dimensional structure (bottom).

**Table S4.** Experimental (SCXRD) and calculated (PBEsol and rSCAN) bond lengths in [K($XeF_2$)$_2$$PtF_6$], the corresponding differences, and calculated bond valences (BV) and bond-valence sum for $K^+$.

| Bond | Experimental (Å) | BV (vu)[a] | PBEsol-TS (Å) | Δ (%)[b] | rSCAN-D4 (Å) | Δ (%)[b] |
|---|---|---|---|---|---|---|
| Xe1−F1 | 1.9829(10) | | 2.023 | 2.0 | 2.016 | 1.7 |
| Xe1−F1$^{iv}$ | 1.9829(10) | | 2.023 | 2.0 | 2.016 | 1.7 |
| Pt1−F2$^{i}$ | 1.8765(18) | | 1.900 | 1.3 | 1.882 | 0.3 |
| Pt1−F2 | 1.8766(18) | | 1.900 | 1.2 | 1.882 | 0.3 |
| Pt1−F3$^{ii}$ | 1.8787(9) | | 1.895 | 0.9 | 1.897 | 1.0 |
| Pt1−F3$^{iii}$ | 1.8787(9) | | 1.895 | 0.9 | 1.897 | 1.0 |
| Pt1−F3 | 1.8787(9) | | 1.895 | 0.9 | 1.897 | 1.0 |
| Pt1−F3$^{i}$ | 1.8787(9) | | 1.895 | 0.9 | 1.897 | 1.0 |
| K1−F3$^{v}$ | 2.7542(9) | 0.127 | 2.736 | −0.7 | 2.732 | −0.8 |
| K1−F3$^{vi}$ | 2.7542(9) | 0.127 | 2.736 | −0.7 | 2.732 | −0.8 |
| K1−F3$^{vii}$ | 2.7542(9) | 0.127 | 2.736 | −0.7 | 2.732 | −0.8 |
| K1−F3 | 2.7542(9) | 0.127 | 2.736 | −0.7 | 2.732 | −0.8 |
| K1−F1$^{viii}$ | 2.6121(10) | 0.187 | 2.601 | −0.4 | 2.574 | −1.5 |
| K1−F1$^{v}$ | 2.6121(10) | 0.187 | 2.601 | −0.4 | 2.574 | −1.5 |
| K1−F1 | 2.6121(10) | 0.187 | 2.601 | −0.4 | 2.574 | −1.5 |
| K1−F1$^{ix}$ | 2.6121(10) | 0.187 | 2.601 | −0.4 | 2.574 | −1.5 |
| | **SUM** | **1.258** | | | | |

[a] Bond-valence parameters[1]: $R_0$ = 1.992 Å, $B$ = 0.37 Å

[b] Calculated as: $\Delta = ((d_{calc} - d_{exp})/d_{exp}) \times 100$

Symmetry codes: (i) −*x* + 1, −*y* + 1, −*z* + 1; (ii) −*y* + 1, *x*, *z*; (iii) *y*, −*x* + 1, −*z* + 1; (iv) −*x* + 3/2, −*y* + 3/2, −*z* + 1/2; (v) −*x* + 1, −*y* + 2, −*z* + 1; (vi) *y* − 1/2, *x* + 1/2, −*z* + 1; (vii) −*y* + 3/2, −*x* + 3/2, *z*; (viii) −*x* + 1, −*y* + 2, *z*; (ix) *x*, *y*, −*z* + 1.

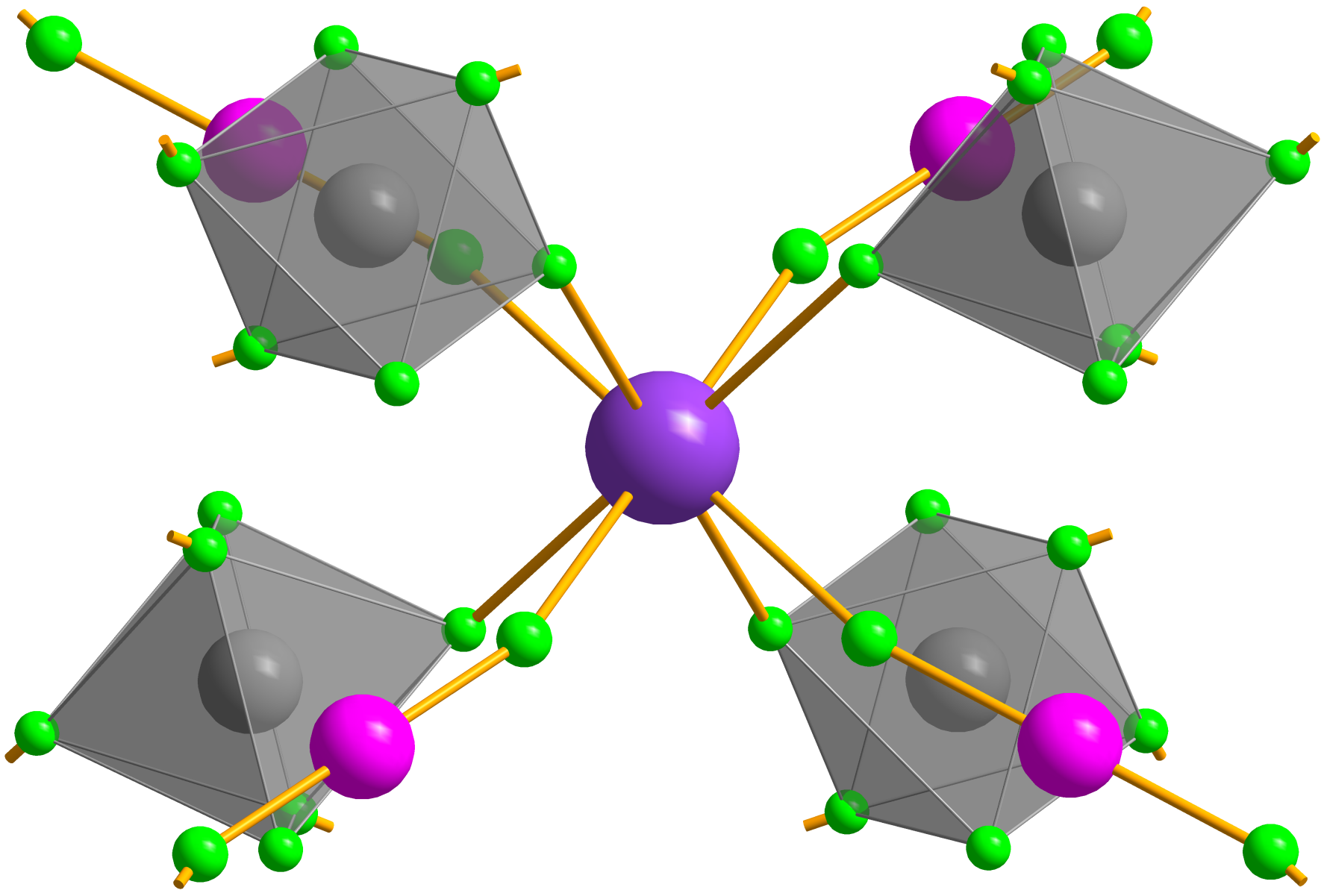

**Figure S3.** Alternative view of the eight-coordinate $K^+$ cation in the crystal structure of $[K(XeF_2)_2PtF_6]$, highlighting its distorted cube-like coordination geometry.

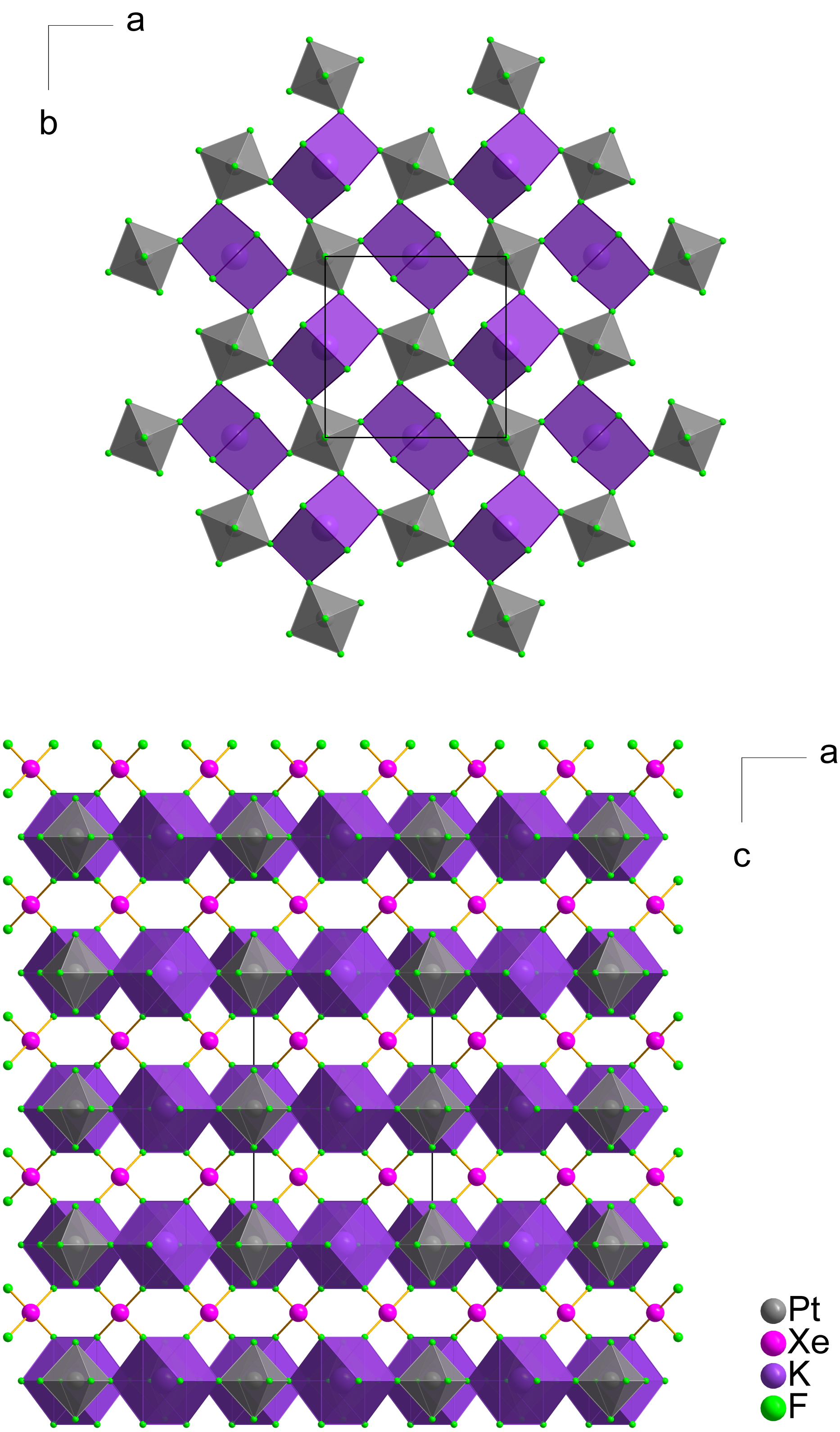


**Figure S4.** Section of the crystal structure of $[K(XeF_2)_2PtF_6]$ showing a layer composed of $[PtF_6]$ and $[KF_8]$ units ($XeF_2$ ligands omitted for clarity) lying parallel to the *ab* plane (top), and the interconnection of these layers into a three-dimensional structure by bridging $XeF_2$ ligands (bottom).

**Table S5.** Hirshfeld atomic charges (e) in [$Li(XeF_2)_3PtF_6$], calculated at the PBEsol-TS and rSCAN-D4 levels of theory.

| Atom | PBEsol-TS | rSCAN-D4 | Atom | PBEsol-TS | rSCAN-D4 |
|---|---|---|---|---|---|
| Pt1 | 1.50 | 1.62 | F8 | −0.55 | −0.56 |
| Pt2 | 1.50 | 1.61 | F9 | −0.54 | −0.56 |
| Xe1 | 1.09 | 1.11 | F10 | −0.54 | −0.56 |
| Xe2 | 1.15 | 1.17 | F11 | −0.54 | −0.55 |
| Xe3 | 1.16 | 1.18 | F12 | −0.55 | −0.56 |
| Xe4 | 1.14 | 1.16 | F13 | −0.43 | −0.44 |
| Xe5 | 1.15 | 1.18 | F14 | −0.41 | −0.42 |
| Xe6 | 1.16 | 1.18 | F15 | −0.43 | −0.48 |
| Li1 | 0.93 | 0.97 | F16 | −0.40 | −0.41 |
| Li2 | 0.93 | 0.94 | F17 | −0.43 | −0.44 |
| F1 | −0.45 | −0.47 | F18 | −0.58 | −0.63 |
| F2 | −0.57 | −0.58 | F19 | −0.41 | −0.42 |
| F3 | −0.54 | −0.55 | F20 | −0.43 | −0.45 |
| F4 | −0.55 | −0.56 | F21 | −0.43 | −0.45 |
| F5 | −0.54 | −0.55 | F22 | −0.43 | −0.45 |
| F6 | −0.55 | −0.57 | F23 | −0.43 | −0.45 |
| F7 | −0.53 | −0.54 | F24 | −0.43 | −0.45 |
| | | | | | |
| Σ($Xe1F_2$) | 0.07 | 0.06 | Σ($Xe4F_2$) | 0.06 | 0.06 |
| Σ($Xe2F_2$) | 0.06 | 0.06 | Σ($Xe5F_2$) | 0.07 | 0.06 |
| Σ($Xe3F_2$) | 0.07 | 0.06 | Σ($Xe6F_2$) | 0.07 | 0.07 |

**Table S6.** Hirshfeld atomic charges (e) in [$Na(XeF_2)PtF_6$], calculated at the PBEsol-TS and rSCAN-D4 levels of theory.

| Atom | PBEsol-TS | rSCAN-D4 |
|---|---|---|
| Pt1 | 1.52 | 1.65 |
| Xe1 | 1.15 | 1.17 |
| Na1 | 1.04 | 1.05 |
| F1 | −0.51 | −0.52 |
| F2 | −0.53 | −0.54 |
| F3 | −0.38 | −0.39 |
| F4 | −0.40 | −0.41 |
| F5 | −0.47 | −0.53 |
| F6 | −0.46 | −0.46 |
| F7 | −0.47 | −0.46 |
| F8 | −0.51 | −0.56 |
| | | |
| Σ($XeF_2$) | 0.11 | 0.11 |

**Table S7.** Hirshfeld atomic charges (e) in [$K(XeF_2)_2PtF_6$], calculated at the PBEsol-TS and rSCAN-D4 levels of theory.

| Atom | PBEsol-TS | rSCAN-D4 |
|---|---|---|
| Pt1 | 1.52 | 1.63 |
| Xe1 | 1.10 | 1.12 |
| K1 | 1.09 | 1.09 |
| F1 | −0.52 | −0.53 |
| F2 | −0.48 | −0.47 |
| F3 | −0.45 | −0.48 |
| | | |
| Σ($XeF_2$) | 0.06 | 0.06 |

In an attempt to evaluate the thermodynamics of $XeF_2$ coordination, the reaction energies Δ*E* were calculated assuming a simplified reaction pathway $n XeF_2(s) + MPtF_6(s) \longrightarrow [M(XeF_2)_n PtF_6](s)$ (M = Li, Na, K). The solid-state energies of the components involved ($XeF_2$, alkali hexafluoridoplatinates(V), and the $XeF_2$–alkali metal coordination compounds) were obtained from periodic DFT calculations performed at the PBEsol-TS and rSCAN-D4 levels of theory.

**Table S8.** Calculated energy changes (Δ*E*) for the formation of the three $XeF_2$ coordination compounds calculated at the PBEsol-TS and the rSCAN-D4 levels of theory, expressed in both eV and kJ per mol of the product.

| Reaction | Δ*E* (PBEsol-TS) (eV) | Δ*E* (PBEsol-TS) (kJ/mol product) | Δ*E* (rSCAN-D4) (eV) | Δ*E* (rSCAN-D4) (kJ/mol product) |
|---|---|---|---|---|
| $3XeF_2(s) + LiPtF_6(s) \longrightarrow [Li(XeF_2)_3PtF_6](s)$ | 0.16 | 15.3 | 0.09 | 8.8 |
| $XeF_2(s) + NaPtF_6(s) \longrightarrow [Na(XeF_2)PtF_6](s)$ | 0.12 | 11.5 | −0.02 | −1.9 |
| $2XeF_2(s) + KPtF_6(s) \longrightarrow [K(XeF_2)_2PtF_6](s)$ | 0.55 | 52.9 | 0.14 | 13.1 |

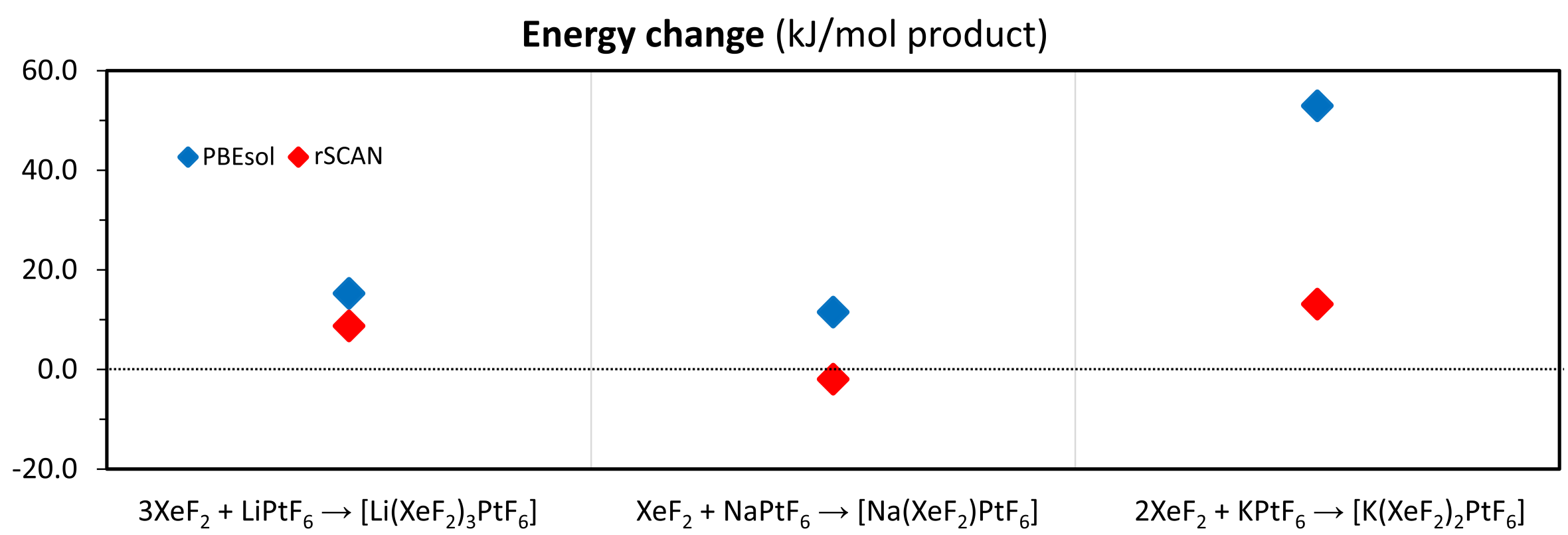


**Figure S5.** Plot of the energy changes (Δ*E*) calculated at the PBEsol-TS and rSCAN-D4 levels of theory for the formation of the three coordination compounds.

The calculations yielded positive Δ*E* values for all reactions except the formation of $[Na(XeF_2)PtF_6]$ at the rSCAN-D4 level of theory. It is important to note that the calculated Δ*E* values do not include zero-point vibrational or finite-temperature contributions. Moreover, in most cases, the calculated energy changes are relatively small ($|\Delta E| < 15$ kJ $mol^{-1}$). Consequently, these calculations alone do not allow the thermodynamic stability of the $[M(XeF_2)_n PtF_6]$ compounds to be assessed reliably. It should also be noted that the reaction pathways considered do not correspond directly to those employed experimentally for the synthesis of the adducts, as they do not account for the influence of the aHF solvent. Nevertheless, at both levels of theory, the reaction yielding $[K(XeF_2)_2PtF_6]$ exhibits the highest Δ*E* value, consistent with the experimental observation that this compound is the least stable of the three complexes and readily decomposes into $XeF_2$ and $KPtF_6$.

**Table S9**. Crystallographic data and refinement results for $LiPtF_6$, $NaPtF_6$, $KPtF_6$, and $RbPtF_6$.

| Compound | $LiPtF_6$ | $NaPtF_6$ | $KPtF_6$ | $RbPtF_6$ |
|---|---|---|---|---|
| Formula | $LiPtF_6$ | $NaPtF_6$ | $KPtF_6$ | $RbPtF_6$ |
| $F_w$ | 316.03 | 332.08 | 348.19 | 394.56 |
| $T$ (K) | 100.00(10) | 99.99(10) | 100.00(10) | 99.99(10) |
| Crystal system | Trigonal | Trigonal | Trigonal | Trigonal |
| Space group | $R\bar{3}$ | $R\bar{3}$ | $R\bar{3}$ | $R\bar{3}$ |
| $a$ (Å) | 4.97492(11) | 5.16973(7) | 7.41013(13) | 7.55677(11) |
| $b$ (Å) | 4.97492(11) | 5.16973(7) | 7.41013(13) | 7.55677(11) |
| $c$ (Å) | 13.5916(3) | 14.7717(2) | 7.28292(15) | 7.54000(12) |
| α (°) | 90 | 90 | 90 | 90 |
| β (°) | 90 | 90 | 90 | 90 |
| γ (°) | 120 | 120 | 120 | 120 |
| $V$ (Å$^3$) | 291.321(15) | 341.898(10) | 346.329(14) | 372.884(13) |
| $Z$ | 3 | 3 | 3 | 3 |
| $\rho_{calc}$ (g/cm$^3$) | 5.404 | 4.839 | 5.008 | 5.271 |
| Crystal size (mm) | 0.14 × 0.12 × 0.06 | 0.07 × 0.07 × 0.06 | 0.14 × 0.132 × 0.102 | 0.109 × 0.086 × 0.059 |
| Radiation type | Ag $K\alpha$ | Ag $K\alpha$ | Ag $K\alpha$ | Ag $K\alpha$ |
| λ (Å) | 0.56087 | 0.56087 | 0.56087 | 0.56087 |
| μ (mm$^{-1}$) | 19.636 | 16.782 | 16.977 | 20.585 |
| $F$(000) | 405 | 429 | 453 | 507 |
| $\theta_{max}$ (°) | 36.450 | 35.3660 | 36.8060 | 36.626 |
| Index ranges | $-10 \le h \le 10$ | $-10 \le h \le 10$ | $-15 \le h \le 15$ | $-15 \le h \le 15$ |
| | $-10 \le k \le 10$ | $-10 \le k \le 10$ | $-15 \le k \le 15$ | $-15 \le k \le 15$ |
| | $-28 \le l \le 27$ | $-30 \le l \le 31$ | $-15 \le l \le 15$ | $-15 \le l \le 15$ |
| Reflections collected | 7834 | 17232 | 9548 | 10146 |
| Independent reflections | 635 | 756 | 760 | 802 |
| Reflections with [$I > 2\sigma(I)$] | 635 | 756 | 760 | 802 |
| $R_{int}$ | 0.0847 | 0.0949 | 0.0879 | 0.0958 |
| $R_{sigma}$ | 0.0268 | 0.0212 | 0.0262 | 0.0277 |
| Data/restraints/parameters | 635/0/15 | 756/0/14 | 760/0/14 | 802/0/15 |
| $S$ | 1.070 | 1.106 | 1.060 | 1.071 |
| $R_1$, $wR_2$ [$I > 2\sigma(I)$] | 0.0192, 0.0462 | 0.0178, 0.0413 | 0.0183, 0.0427 | 0.0181, 0.0436 |
| $R_1$, $wR_2$ [all data] | 0.0192, 0.0462 | 0.0178, 0.0413 | 0.0183, 0.0427 | 0.0181, 0.0436 |
| $\Delta\rho_{min}$, $\Delta\rho_{max}$ (e/Å$^3$) | −3.113, 2.805 | −2.219, 3.295 | −3.421, 2.138 | −2.411, 2.573 |

**Table S10.** Li−F bond lengths and bond-valence calculations for $Li^+$ in $LiPtF_6$.

| Bond | Length (Å) | BV (vu)[a] |
|---|---|---|
| Li1–F1 | 2.0179(15) | 0.169 |
| Li1–F1$^{i}$ | 2.0179(15) | 0.169 |
| Li1–F1$^{ii}$ | 2.0179(15) | 0.169 |
| Li1–F1$^{iii}$ | 2.0179(15) | 0.169 |
| Li1–F1$^{iv}$ | 2.0179(15) | 0.169 |
| Li1–F1$^{v}$ | 2.0179(15) | 0.169 |
| | SUM | **1.014** |

[a] Bond-valence parameters[1]: $R_0$ = 1.360 Å, $B$ = 0.37 Å

Symmetry codes: (i) –*x*, –*y*, –*z*; (ii) *y*, –*x* + *y*, –*z*; (iii) –*x* + *y*, –*x*, *z*; (iv) –*y*, *x* – *y*, *z*; (v) *x* – *y*, *x*, –*z*.

**Table S11.** Na−F bond lengths and bond-valence calculations for $Na^+$ in $NaPtF_6$.

| Bond | Length (Å) | BV (vu)[a] |
|---|---|---|
| Na1–F1 | 2.2900(13) | 0.191 |
| Na1–F1$^{i}$ | 2.2899(13) | 0.191 |
| Na1–F1$^{ii}$ | 2.2899(13) | 0.191 |
| Na1–F1$^{iii}$ | 2.2899(13) | 0.191 |
| Na1–F1$^{iv}$ | 2.2899(13) | 0.191 |
| Na1–F1$^{v}$ | 2.2899(13) | 0.191 |
| | SUM | **1.145** |

[a] Bond-valence parameters[1]: $R_0$ = 1.677 Å, $B$ = 0.37 Å

Symmetry codes: (i) –*x*, –*y*, –*z*; (ii) *y*, –*x* + *y*, –*z*; (iii) –*x* + *y*, –*x*, *z*; (iv) –*y*, *x* – *y*, *z*; (v) *x* – *y*, *x*, –*z*.

**Table S12.** K–F bond lengths and bond-valence calculations for $K^+$ in $KPtF_6$.

| Bond | Length (Å) | BV (vu)[a] |
|---|---|---|
| K1–F1$^{i}$ | 2.8369(14) | 0.102 |
| K1–F1$^{ii}$ | 2.8369(14) | 0.102 |
| K1–F1$^{iii}$ | 2.8369(14) | 0.102 |
| K1–F1$^{iv}$ | 2.8369(14) | 0.102 |
| K1–F1$^{v}$ | 2.8369(14) | 0.102 |
| K1–F1$^{vi}$ | 2.8369(14) | 0.102 |
| K1–F1 | 2.9367(15) | 0.078 |
| K1–F1$^{vii}$ | 2.9367(15) | 0.078 |
| K1–F1$^{viii}$ | 2.9367(15) | 0.078 |
| K1–F1$^{ix}$ | 2.9367(15) | 0.078 |
| K1–F1$^{x}$ | 2.9367(15) | 0.078 |
| K1–F1$^{xi}$ | 2.9367(15) | 0.078 |
| | SUM | **1.079** |

[a] Bond-valence parameters[1]: $R_0$ = 1.992 Å, $B$ = 0.37 Å

Symmetry codes: (i) $-x + y + 1/3, -x + 1/3, z + 1/3$; (ii) $y - 2/3, -x + y - 1/3, -z + 2/3$; (iii) $x - 1/3, y - 2/3, z + 1/3$; (iv) $-y + 2/3, x - y + 1/3, z + 1/3$; (v) $x - y + 1/3, x - 1/3, -z + 2/3$; (vi) $-x + 1/3, -y + 2/3, -z + 2/3$; (vii) $x - y, x, -z + 1$; (viii) $-y, x - y, z$; (ix) $-x + y, -x, z$; (x) $-x, -y, -z + 1$; (xi) $y, -x + y, -z + 1$.

**Table S13.** Rb−F bond lengths and bond-valence calculations for $Rb^+$ in $RbPtF_6$.

| Bond | Length (Å) | BV (vu)[a] |
|---|---|---|
| Rb1–F1$^{i}$ | 2.9291(12) | 0.125 |
| Rb1–F1$^{ii}$ | 2.9290(13) | 0.125 |
| Rb1–F1$^{iii}$ | 2.9291(12) | 0.125 |
| Rb1–F1$^{iv}$ | 2.9290(12) | 0.125 |
| Rb1–F1$^{v}$ | 2.9290(13) | 0.125 |
| Rb1–F1$^{vi}$ | 2.9290(13) | 0.125 |
| Rb1–F1 | 3.0600(13) | 0.088 |
| Rb1–F1$^{vii}$ | 3.0600(13) | 0.088 |
| Rb1–F1$^{viii}$ | 3.0600(13) | 0.088 |
| Rb1–F1$^{ix}$ | 3.0600(13) | 0.088 |
| Rb1–F1$^{x}$ | 3.0600(13) | 0.088 |
| Rb1–F1$^{xi}$ | 3.0600(13) | 0.088 |
| | **SUM** | **1.277** |

[a] Bond-valence parameters[2]: $R_0$ = 2.16 Å, $B$ = 0.37 Å

Symmetry codes: (i) $-x + y + 1/3, -x + 1/3, z + 1/3$; (ii) $y - 2/3, -x + y - 1/3, -z + 2/3$; (iii) $x - 1/3, y - 2/3, z + 1/3$; (iv) $-y + 2/3, x - y + 1/3, z + 1/3$; (v) $x - y + 1/3, x - 1/3, -z + 2/3$; (vi) $-x + 1/3, -y + 2/3, -z + 2/3$; (vii) $x - y, x, -z + 1$; (viii) $-y, x - y, z$; (ix) $-x + y, -x, z$; (x) $-x, -y, -z + 1$; (xi) $y, -x + y, -z + 1$.

**Table S14.** Calculated energies of the $MPtF_6$ (M = Li, Na, K, Rb) compounds in the $LiSbF_6$- and $KAsF_6$-type structures calculated at the PBEsol-TS and rSCAN-D4 levels of theory.

| | *E* (PBEsol-TS) (eV) | *E* (rSCAN-D4) (eV) | *E* (PBEsol-TS) (eV) | *E* (rSCAN-D4) (eV) |
|---|---|---|---|---|
| | $LiSbF_6$-type structure | | $KAsF_6$-type structure | |
| $LiPtF_6$ | −22969.55 | −22917.16 | | |
| $NaPtF_6$ | −26073.90 | −26025.79 | −26073.70 | −26025.11 |
| $KPtF_6$ | −24767.33 | −24692.58 | −24768.65 | −24693.24 |
| $RbPtF_6$ | −24482.49 | −24398.63 | −24483.93 | −24399.25 |

**Table S15.** Calculated unit-cell volumes of the $MPtF_6$ (M = Li, Na, K, Rb) compounds in the $LiSbF_6$- and $KAsF_6$-type structures calculated at the PBEsol-TS and rSCAN-D4 levels of theory.

| | *V* (PBEsol-TS) (Å$^3$) | *V* (rSCAN-D4) (Å$^3$) | *V* (PBEsol-TS) (Å$^3$) | *V* (rSCAN-D4) (Å$^3$) |
|---|---|---|---|---|
| | $LiSbF_6$-type structure | | $KAsF_6$-type structure | |
| $LiPtF_6$ | 282.44 | 272.38 | | |
| $NaPtF_6$ | 339.20 | 332.37 | 298.17 | 297.46 |
| $KPtF_6$ | 379.61 | 389.64 | 317.94 | 332.46 |
| $RbPtF_6$ | 405.69 | 397.77 | 339.52 | 360.15 |

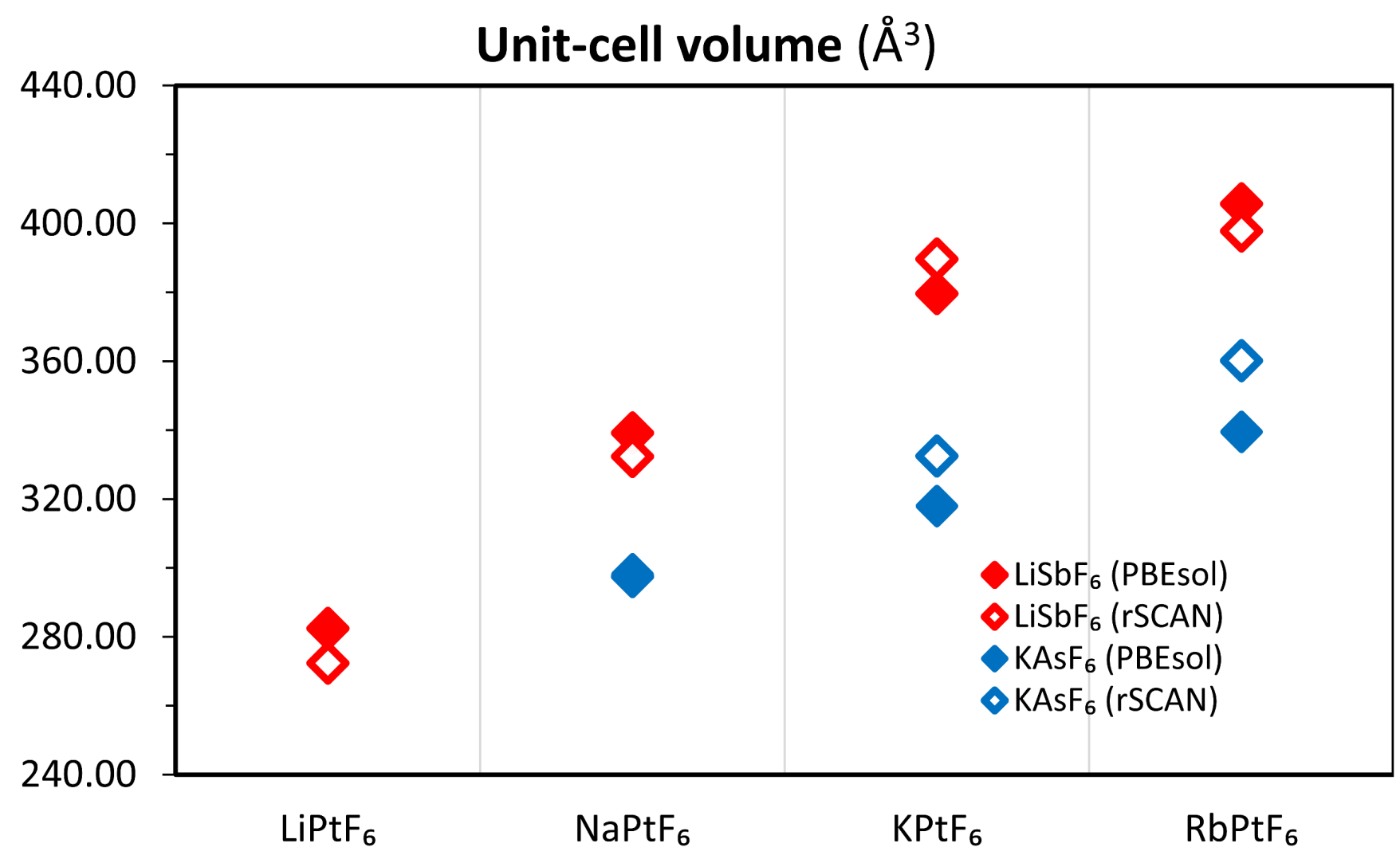


**Figure S6.** Plot comparing the unit-cell volumes calculated for $MPtF_6$ (M = Li, Na, K, Rb) in the $LiSbF_6$ and $KAsF_6$ structure types at the PBEsol-TS and rSCAN-D4 levels of theory.

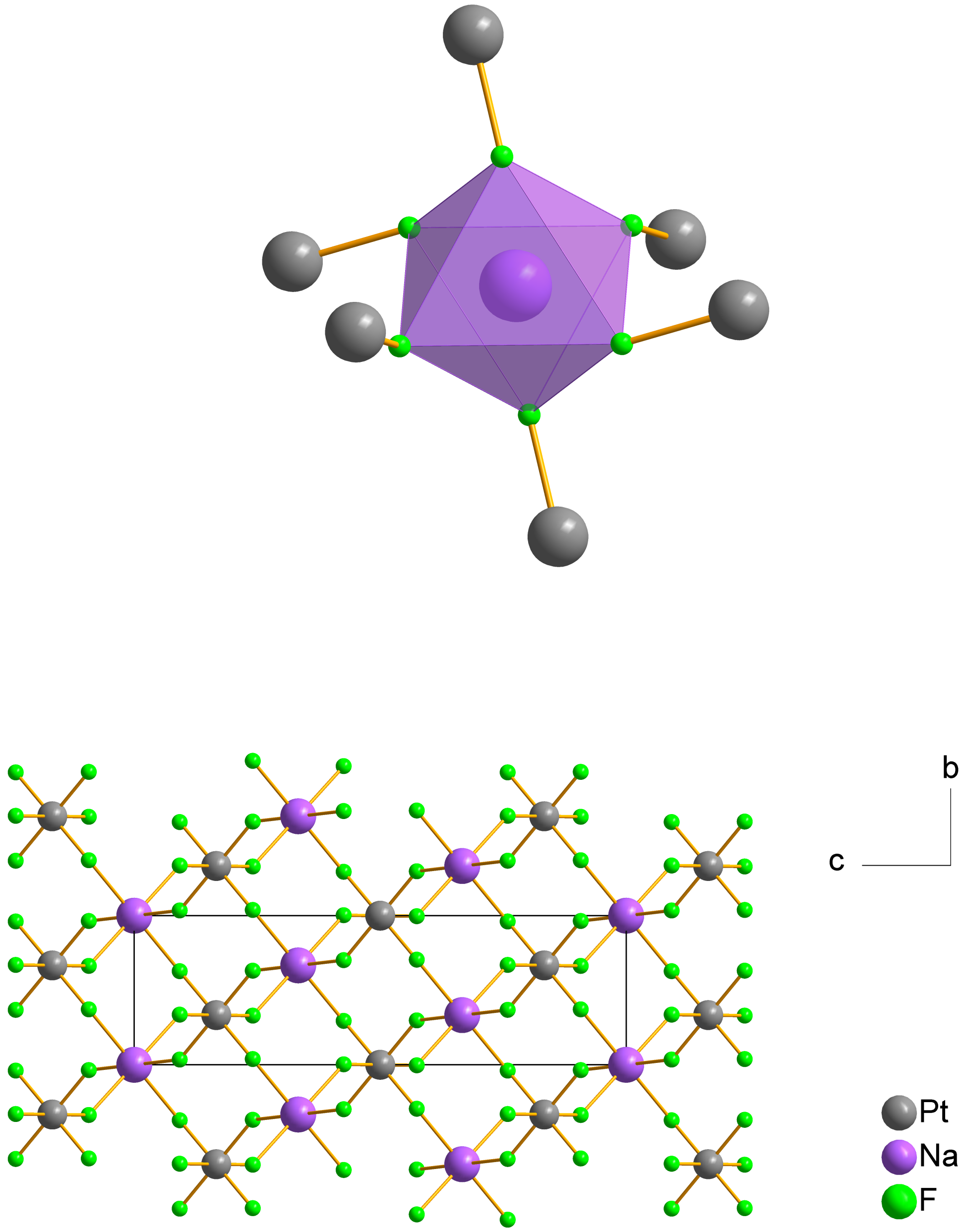


**Figure S7.** Coordination environment of the $Na^+$ cation, illustrating a slightly distorted octahedral geometry (top), and the crystal packing of $NaPtF_6$ viewed along the *a* crystallographic axis (bottom). An analogous arrangement is observed in the crystal structure of $LiPtF_6$.

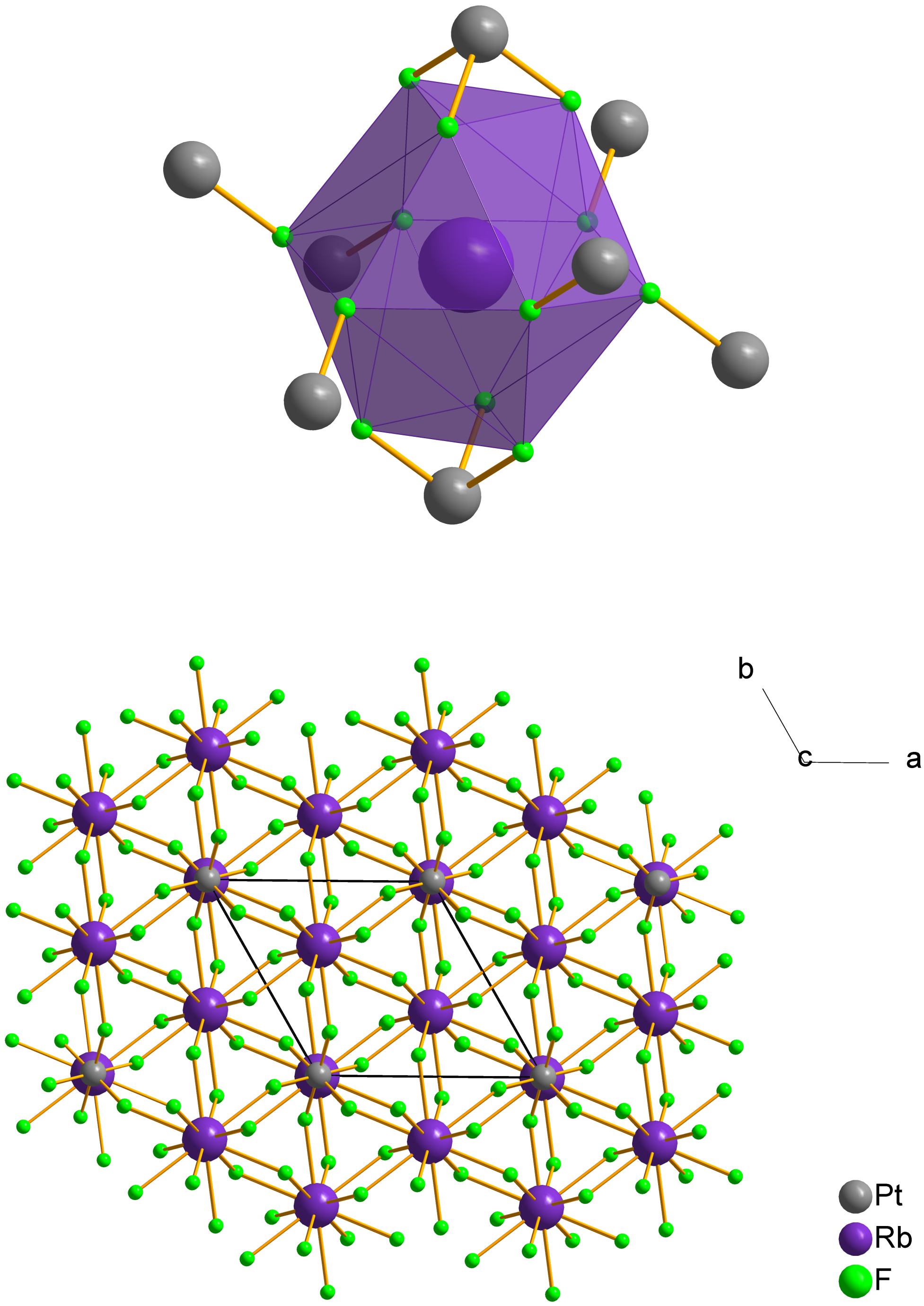


**Figure S8.** Coordination environment of the $Rb^{+}$ cation, illustrating a distorted cuboctahedron geometry (top), and the crystal packing of $RbPtF_6$ viewed along the *c* crystallographic axis (bottom). An analogous arrangement is observed in the crystal structure of $KPtF_6$.

**Table S16.** Observed bands in the low-temperature Raman spectra of [Li($XeF_2$)$_3$$PtF_6$], [Na($XeF_2$)$PtF_6$], and [K($XeF_2$)$_2$$PtF_6$] (Figure 6) and in the room-temperature Raman spectra of powdered samples of $LiPtF_6$, $NaPtF_6$, $KPtF_6$, and $RbPtF_6$ (Figure 7), with relative intensities given in parentheses.

| [Li($XeF_2$)$_3$$PtF_6$] | [Na($XeF_2$)$PtF_6$] | [K($XeF_2$)$_2$$PtF_6$] |
|---|---|---|
| Δν (cm$^{-1}$) | Δν (cm$^{-1}$) | Δν (cm$^{-1}$) |
| 60 (12) | 63 (15) | 57 (23) |
| 72 (17) | 88 (20) | 70 (21) |
| 84 (21) | 217 (3) | 113 (8) |
| 86 sh | 235 (58) | 233 (90) |
| 119 (16) | 242 (37) | 241 (12) |
| 131 (16) | 253 sh | 251 (2) |
| 155 (10) | 496 (2) | 523 (74) |
| 198 (3) | 509 (4) | 527 (30) |
| 229 (56) | 524 (12) | 571 (3) |
| 233 sh | 535 (23) | 583 (15) |
| 497 (10) | 548 (6) | 590 (8) |
| 519 (100) | 578 (11) | 642 (100) |
| 572 (12) | 583 (10) | |
| 577 (9) | 639 (100) | |
| 598 (2) | 659 (34) | |
| 630 (61) | 673 (2) | |
| 643 (16) | | |
| 660 (4) | | |

| $LiPtF_6$ | $NaPtF_6$ | $KPtF_6$ | $RbPtF_6$ |
|---|---|---|---|
| Δν (cm$^{-1}$) | Δν (cm$^{-1}$) | Δν (cm$^{-1}$) | Δν (cm$^{-1}$) |
| 103 (7) | 72 (5) | 237 (23) | 238 (44) |
| 136 (3) | 242 (92) | 250 (92) | 245 (100) |
| 254 (100) | 581 (14) | 574 (19) | 571 (16) |
| 261 (54) | 649 (100) | 641 (100) | 639 (84) |
| 573 (5) | | | |
| 652 (76) | | | |

sh: shoulder

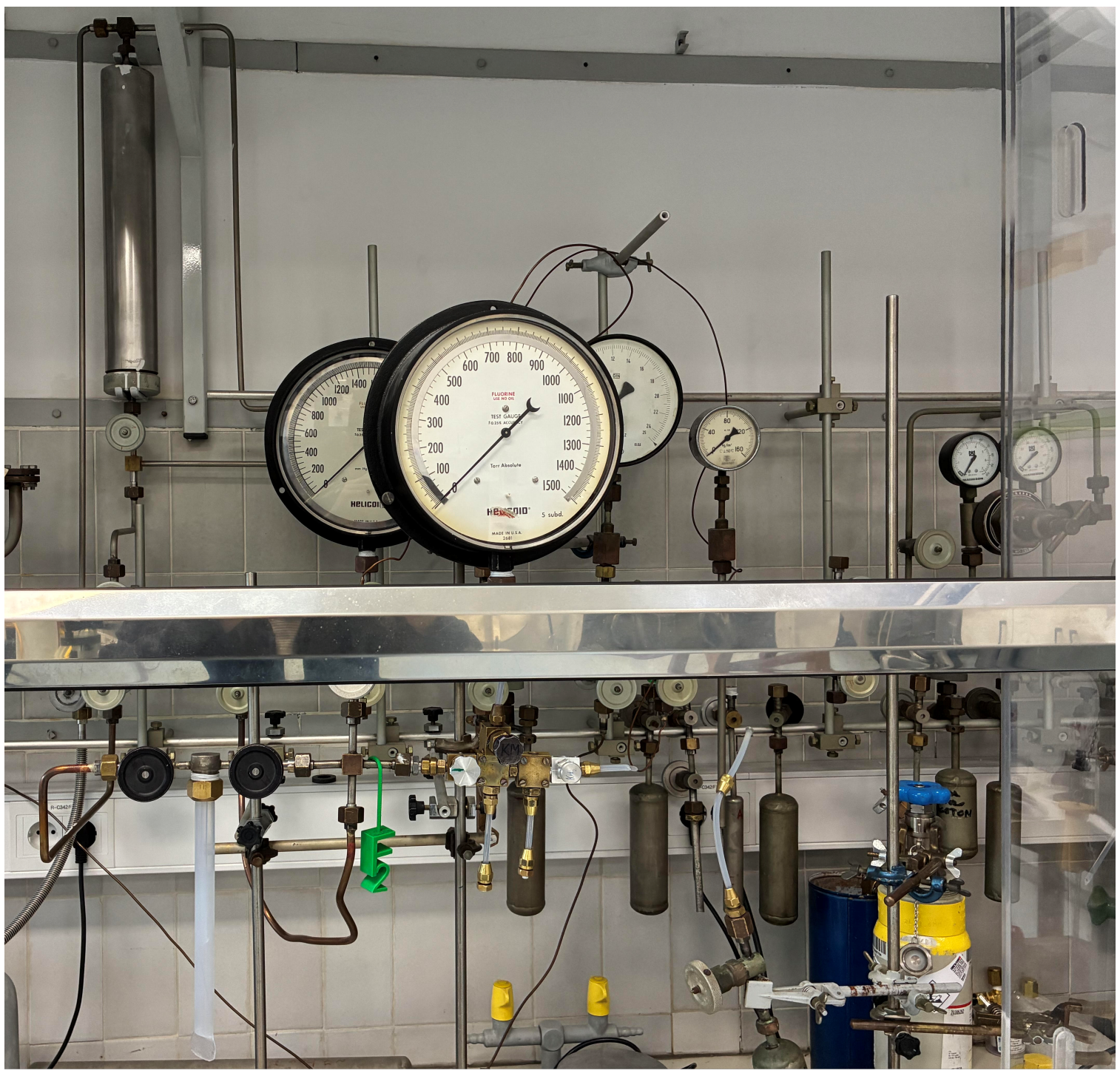

**Figure S9.** Vacuum line constructed from nickel, copper, polytetrafluoroethylene (PTFE), and fluorinated ethylene propylene (FEP) copolymer, used for the addition or removal of volatile species.

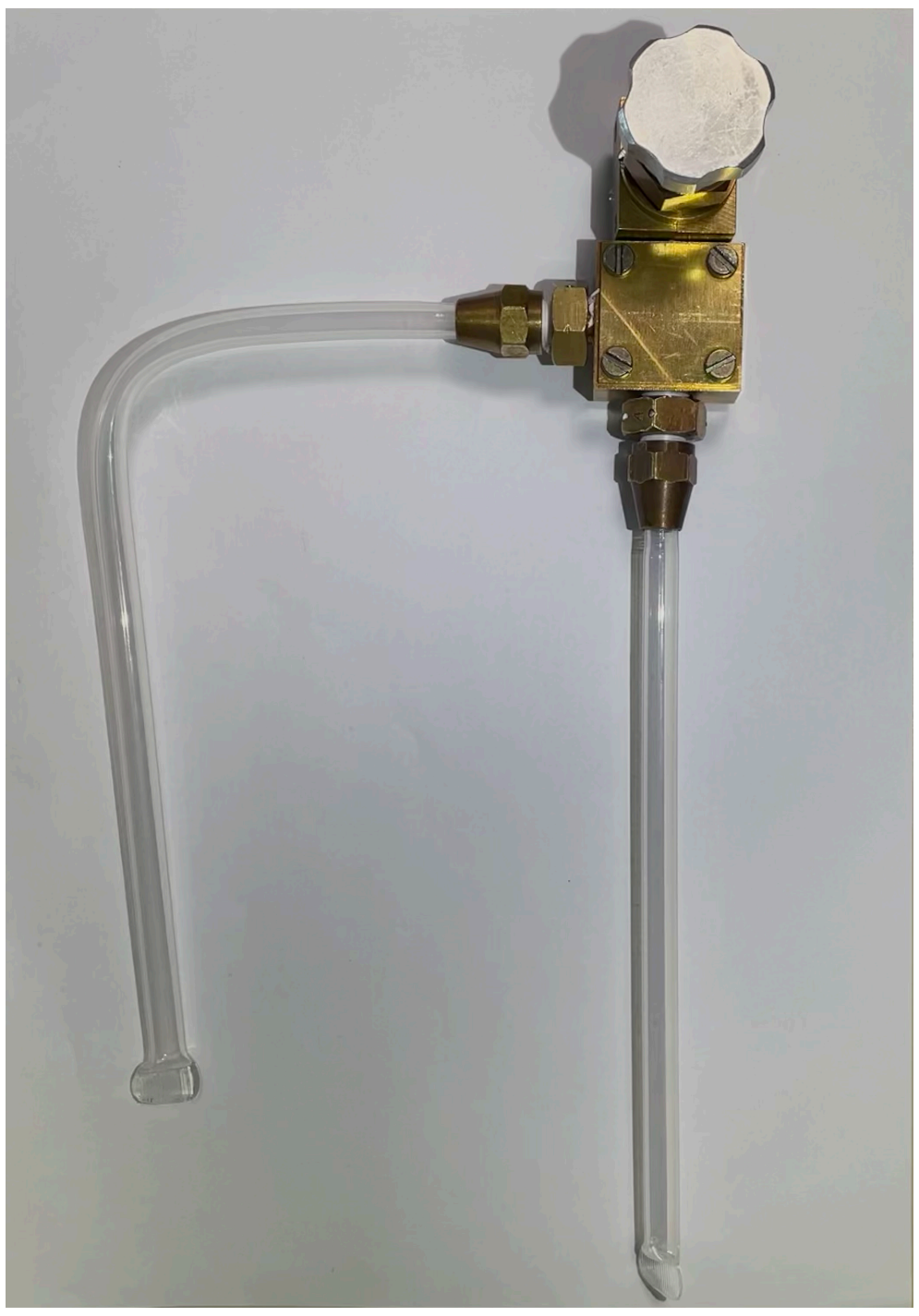

**Figure S10.** The h-shaped vessel, constructed from FEP tubing and brass-encased PTFE components, was used for the reactions described in this study. Throughout the manuscript, the angled arm visible on the left is termed the side arm, whereas the straight arm extending vertically from the T-junction is termed the main arm.

**Table S17.** Atomic coordinates of the DFT-optimized crystal structure of $[Li(XeF_2)_3PtF_6]$

| PBEsol-TS | | | |
|---|---|---|---|
| Atom | *x* | *y* | *z* |
| Pt1 | 0.673957 | 0.404932 | 0.777913 |
| Pt2 | 0.726655 | 0.647814 | 0.810525 |
| Xe1 | 0.175413 | 0.388322 | 0.254573 |
| Xe2 | 0.256733 | 0.268588 | 0.669502 |
| Xe3 | 0.696120 | 0.518047 | 0.278144 |
| Xe4 | 0.749266 | 0.268019 | 0.165314 |
| Xe5 | 0.219925 | 0.644595 | 0.306701 |
| Xe6 | 0.192179 | 0.518906 | 0.779386 |
| Li1 | 0.195668 | 0.516838 | 0.280749 |
| Li2 | 0.755933 | 0.274219 | 0.665250 |
| F1 | 0.231879 | 0.322772 | 0.128747 |
| F2 | 0.110783 | 0.454416 | 0.389105 |
| F3 | 0.517611 | 0.238341 | 0.762151 |
| F4 | 0.000271 | 0.300719 | 0.569687 |
| F5 | 0.435257 | 0.487022 | 0.189032 |
| F6 | 0.956531 | 0.549590 | 0.368523 |
| F7 | 0.615354 | 0.287027 | 0.414174 |
| F8 | 0.884554 | 0.250311 | 0.914911 |
| F9 | 0.288116 | 0.580394 | 0.167453 |
| F10 | 0.152120 | 0.708890 | 0.445050 |
| F11 | 0.063661 | 0.498724 | 0.030007 |
| F12 | 0.323278 | 0.539370 | 0.529524 |
| F13 | 0.691892 | 0.404559 | 0.055691 |
| F14 | 0.656779 | 0.404662 | 0.500536 |
| F15 | 0.681947 | 0.475155 | 0.774981 |
| F16 | 0.950844 | 0.402615 | 0.777103 |
| F17 | 0.396562 | 0.406601 | 0.779905 |
| F18 | 0.661000 | 0.333245 | 0.783410 |
| F19 | 0.717414 | 0.577596 | 0.811445 |
| F20 | 0.711473 | 0.648162 | 0.532842 |
| F21 | 0.741925 | 0.647665 | 0.088270 |
| F22 | 0.449268 | 0.650780 | 0.810618 |
| F23 | 0.004075 | 0.644985 | 0.810587 |
| F24 | 0.735807 | 0.718217 | 0.809599 |

| rSCAN-D4 | | | |
|---|---|---|---|
| Atom | *x* | *y* | *z* |
| Pt1 | 0.674192 | 0.404610 | 0.777776 |
| Pt2 | 0.726545 | 0.647944 | 0.810586 |
| Xe1 | 0.174797 | 0.388244 | 0.255194 |
| Xe2 | 0.255589 | 0.268834 | 0.668522 |
| Xe3 | 0.695806 | 0.518141 | 0.277969 |
| Xe4 | 0.749799 | 0.268367 | 0.165023 |
| Xe5 | 0.220366 | 0.644672 | 0.307308 |
| Xe6 | 0.191427 | 0.519090 | 0.779157 |
| Li1 | 0.195544 | 0.517199 | 0.280254 |
| Li2 | 0.755130 | 0.273671 | 0.665374 |
| F1 | 0.232070 | 0.321948 | 0.130328 |
| F2 | 0.110351 | 0.455153 | 0.388783 |
| F3 | 0.520478 | 0.238364 | 0.758494 |
| F4 | −0.005496 | 0.301047 | 0.571365 |
| F5 | 0.431249 | 0.486861 | 0.190575 |
| F6 | 0.960602 | 0.549392 | 0.367027 |
| F7 | 0.616424 | 0.287326 | 0.416727 |
| F8 | 0.884445 | 0.250539 | 0.911780 |
| F9 | 0.286333 | 0.579582 | 0.168655 |
| F10 | 0.154919 | 0.709824 | 0.445262 |
| F11 | 0.063642 | 0.498866 | 0.032857 |
| F12 | 0.322428 | 0.539068 | 0.526036 |
| F13 | 0.690334 | 0.404266 | 0.057432 |
| F14 | 0.656917 | 0.404416 | 0.498594 |
| F15 | 0.681496 | 0.475903 | 0.775486 |
| F16 | 0.953282 | 0.402610 | 0.778181 |
| F17 | 0.394744 | 0.406027 | 0.779435 |
| F18 | 0.662384 | 0.331952 | 0.782197 |
| F19 | 0.718161 | 0.577274 | 0.811019 |
| F20 | 0.711354 | 0.648360 | 0.529662 |
| F21 | 0.741562 | 0.647929 | 0.091428 |
| F22 | 0.445891 | 0.650471 | 0.810486 |
| F23 | 0.007254 | 0.645303 | 0.811087 |
| F24 | 0.734938 | 0.718841 | 0.810996 |

**Table S18.** Atomic coordinates of the DFT-optimized crystal structure of [Na($XeF_2$)$PtF_6$]

| PBEsol-TS | | | |
|---|---|---|---|
| Atom | *x* | *y* | *z* |
| Pt1 | 0.287159 | 0.893433 | 0.702387 |
| Xe1 | 0.200449 | 0.312881 | 0.514584 |
| Na1 | 0.224166 | 0.563148 | 0.821309 |
| F1 | 0.213682 | 0.139777 | 0.416961 |
| F2 | 0.181632 | 0.487500 | 0.611689 |
| F3 | 0.368428 | 0.935394 | 0.877227 |
| F4 | 0.460798 | 0.751439 | 0.709060 |
| F5 | 0.462888 | 0.016139 | 0.643601 |
| F6 | 0.205155 | 0.848593 | 0.526487 |
| F7 | 0.110935 | 0.035522 | 0.696418 |
| F8 | 0.109651 | 0.770426 | 0.760418 |

| rSCAN-D4 | | | |
|---|---|---|---|
| Atom | *x* | *y* | *z* |
| Pt1 | 0.287118 | 0.895192 | 0.701638 |
| Xe1 | 0.197816 | 0.312328 | 0.513846 |
| Na1 | 0.225464 | 0.562961 | 0.819321 |
| F1 | 0.207237 | 0.136301 | 0.417621 |
| F2 | 0.182817 | 0.489840 | 0.610127 |
| F3 | 0.370492 | 0.937741 | 0.876918 |
| F4 | 0.460936 | 0.752752 | 0.708310 |
| F5 | 0.464389 | 0.020587 | 0.642063 |
| F6 | 0.204615 | 0.849667 | 0.525233 |
| F7 | 0.110042 | 0.037321 | 0.695125 |
| F8 | 0.108188 | 0.769357 | 0.761014 |

**Table S19.** Atomic coordinates of the DFT-optimized crystal structure of $[K(XeF_2)_2PtF_6]$

| PBEsol-TS | | | |
|---|---|---|---|
| Atom | *x* | *y* | *z* |
| Pt1 | 0.500000 | 0.500000 | 0.500000 |
| Xe1 | 0.750000 | 0.750000 | 0.250000 |
| K1 | 0.500000 | 0.000000 | 0.500000 |
| F1 | 0.619206 | 0.880794 | 0.340694 |
| F2 | 0.500000 | 0.500000 | 0.358921 |
| F3 | 0.412343 | 0.698790 | 0.500000 |

| rSCAN-D4 | | | |
|---|---|---|---|
| Atom | *x* | *y* | *z* |
| Pt1 | 0.500000 | 0.500000 | 0.500000 |
| Xe1 | 0.750000 | 0.750000 | 0.250000 |
| K1 | 0.500000 | 0.000000 | 0.500000 |
| F1 | 0.617787 | 0.882213 | 0.339698 |
| F2 | 0.500000 | 0.500000 | 0.358153 |
| F3 | 0.411152 | 0.698966 | 0.500000 |

**Table S20.** Atomic coordinates of the DFT-optimized crystal structure of $LiPtF_6$

| PBEsol-TS | | | |
|---|---|---|---|
| Atom | *x* | *y* | *z* |
| Pt1 | 0.000000 | 0.000000 | 0.000000 |
| Li1 | 0.000000 | 0.000000 | 0.500000 |
| F1 | 0.656676 | 0.969343 | 0.585305 |

| rSCAN-D4 | | | |
|---|---|---|---|
| Atom | *x* | *y* | *z* |
| Pt1 | 0.000000 | 0.000000 | 0.000000 |
| Li1 | 0.000000 | 0.000000 | 0.500000 |
| F1 | 0.659601 | 0.970823 | 0.583901 |

**Table S21.** Atomic coordinates of the DFT-optimized crystal structure of $NaPtF_6$

| PBEsol-TS | | | |
|---|---|---|---|
| Atom | *x* | *y* | *z* |
| Pt1 | 0.000000 | 0.000000 | 0.000000 |
| Na1 | 0.000000 | 0.000000 | 0.500000 |
| F1 | 0.340081 | 0.371225 | 0.592130 |

| rSCAN-D4 | | | |
|---|---|---|---|
| Atom | *x* | *y* | *z* |
| Pt1 | 0.000000 | 0.000000 | 0.000000 |
| Na1 | 0.000000 | 0.000000 | 0.500000 |
| F1 | 0.341524 | 0.372111 | 0.590994 |

**Table S22.** Atomic coordinates of the DFT-optimized crystal structure of $KPtF_6$

| PBEsol-TS | | | |
|---|---|---|---|
| Atom | *x* | *y* | *z* |
| Pt1 | 0.000000 | 0.000000 | 0.000000 |
| K1 | 0.000000 | 0.000000 | 0.500000 |
| F1 | 0.838776 | 0.568646 | 0.492458 |

| rSCAN-D4 | | | |
|---|---|---|---|
| Atom | *x* | *y* | *z* |
| Pt1 | 0.000000 | 0.000000 | 0.000000 |
| K1 | 0.000000 | 0.000000 | 0.500000 |
| F1 | 0.837741 | 0.565109 | 0.489571 |

**Table S23.** Atomic coordinates of the DFT-optimized crystal structure of $RbPtF_6$

| PBEsol-TS | | | |
|---|---|---|---|
| Atom | *x* | *y* | *z* |
| Pt1 | 0.000000 | 0.000000 | 0.000000 |
| Rb1 | 0.000000 | 0.000000 | 0.500000 |
| F1 | 0.842924 | 0.563523 | 0.485918 |

| rSCAN-D4 | | | |
|---|---|---|---|
| Atom | *x* | *y* | *z* |
| Pt1 | 0.000000 | 0.000000 | 0.000000 |
| Rb1 | 0.000000 | 0.000000 | 0.500000 |
| F1 | 0.840429 | 0.559000 | 0.482699 |

## References


(1) Brown, I. D.; Altermatt, D. Bond−Valence Parameters Obtained from a Systematic Analysis of the Inorganic Crystal Structure Database. *Acta Crystallogr. B* **1985**, *41* (4), 244–247. https://doi.org/10.1107/S0108768185002063.

(2) Brese, N. E.; O'Keeffe, M. Bond−Valence Parameters for Solids. *Acta Crystallogr. B* **1991**, *47* (2), 192–197. https://doi.org/10.1107/S0108768190011041.
